# A Hamilton principle-based model for diffusion-driven biofilm growth

**DOI:** 10.1007/s10237-024-01883-x

**Published:** 2024-09-30

**Authors:** Felix Klempt, Meisam Soleimani, Peter Wriggers, Philipp Junker

**Affiliations:** Institue of Continuum Mechanics, Leibniz University Hannover, An der Universität 1, 30823 Garbsen, Lower Saxony Germany

**Keywords:** Growth, Biofilm, Hamilton principle, Multi-physics, Finite Element Simulation

## Abstract

Dense communities of bacteria, also known as biofilms, are ubiquitous in all of our everyday life. They are not only always surrounding us, but are also active inside our bodies, for example in the oral cavity. While some biofilms are beneficial or even necessary for human life, others can be harmful. Therefore, it is highly important to gain an in-depth understanding of biofilms which can be achieved by *in vitro* or *in vivo* experiments. Since these experiments are often time-consuming or expensive, *in silico* models have proven themselves to be a viable tool in assisting the description and analysis of these complicated processes. Current biofilm growth simulations are using mainly two approaches for describing the underlying models. The volumetric approach splits the deformation tensor into a growth and an elastic part. In this approach, the mass never changes, unless some additional constraints are enforced. The density-based approach, on the other hand, uses an evolution equation to update the growing tissue by adding mass. Here, the density stays constant, and no pressure is exerted. The *in silico* model presented in this work combines the two approaches. Thus, it is possible to capture stresses inside of the biofilm while adding mass. Since this approach is directly derived from Hamilton’s principle, it fulfills the first and second law of thermodynamics automatically, which other models need to be checked for separately. In this work, we show the derivation of the model as well as some selected numerical experiments. The numerical experiments show a good phenomenological agreement with what is to be expected from a growing biofilm. The numerical behavior is stable, and we are thus capable of solving complicated boundary value problems. In addition, the model is very reactive to different input parameters, thereby different behavior of various biofilms can be captured without modifying the model.

## Introduction

With an estimated half of the biomass in the world consisting of prokaryotes living in every biome of the planet, one could say that microorganisms are the most successful forms of life (Flemming and Wingender, 2010). Most of the time, these microorganisms live in diverse communities attached to an interface between two phases (e.g., solid–liquid). In literature, it is widely agreed to call this arrangement biofilm (Böl et al. 2013). These biofilms do not only consist of multiple species of microorganisms, but also of a self-secreted extracellular polymeric substance (EPS), water, a substratum and nutrients. EPS composes itself of polysaccharides, proteins, DNA, and lipids and represent of more than 90$\%$ of the entire organic matter inside a biofilm (Nielsen et al. 1997; Flemming and Wingender 2010). Because of the high volume fraction of EPS, it is subject to most of the mechanical stresses inside of the biofilm (Billings et al. 2015).

Due to their abundant nature, biofilms have an enormous impact on human life for better or for worse. For instance, beneficial biofilms are used in industrial applications like water treatment units (Soleimani et al. 2016). Harmful ones can be found on implant surfaces among other things where they can be detrimental to the surrounding bone structure. In addition, over 80$\%$ of microbial infections can be traced back to biofilms (Klapper & Dockery, 2010).

Biofilm formation can roughly be divided into four steps. At first, a conditioning film is established (Soleimani 2017). Organic material starts to accumulate at the interface between the two phases. In this step, not only the material of which the substrate is composed is important, but also the microstructure. It is shown that on rougher surfaces, more microorganisms can adhere (Chinnaraj et al. 2021). In the second step, planktonic microorganisms attach to the preconditioned surface. This attachment is considered reversible since the biofilm can be detached by rinsing the surface with water. The main forces acting here are van der Waals forces. After the initial attachment, EPS is produced which strengthen the currently irreversible bond between the substratum and the biofilm. In this phase, the main biofilm growth happens which is focus of this work. After maturation, parts of the biofilm detach. This can be part of a survival strategy to expand to places with more nutrition or it could be due to mechanical stresses on the biofilm. Either way, the growth and the detachment balance each other out, such that an equilibrium height in order of micrometer is achieved. The exact height of the equilibrium depends on numerous factors, including nutrient supply and flow velocity as well as the species involved (Albero et al. 2014).

Growth in this context is defined by a change in mass and microstructure. Not only cell division performed by the microorganisms, but also their production of EPS is an important factor of biofilm growth. The change in microstructure itself without a volume change is called remodeling. Harder biological structures like bones and teeth tend to remodel, whereas soft tissues tend to grow (Albero et al., 2018).

In order to gain a better understanding of the growth and remodeling process, models have proven themselves to be a viable tool in analyzing complicated processes (Chaudhry and Beg, 1998). These models are based on a mathematical description, which requires careful attention, since growth has to be modeled in the framework of an open system in which the local mass produced has to be taken into account (Soleimani et al. 2020). Mass added by growth can be regarded as a compliant or non-compliant source of momentum (Goriely 2017). Furthermore, the second law of thermodynamics should not be violated. The thermodynamics and mechanics of growth are discussed in detail by Epstein and Maugin (2000) and Lubarda and Hoger (2002). Since growth happens orders of magnitude slower than nutrient transport or fluid flow, a common assumption is that the faster processes have already reached a steady state when dealing with the slower processes. This practice is called time homogenization (Haouala & Doghri, 2015). On the other hand, the diverse structure already implies that biofilms are non-homogeneous which adds other difficulties in describing them. Despite the difficulties of implementing, mathematical models offer useful features in investigating biofilms. The development and morphology of the biofilm can be predicted in a wide range of environmental conditions (Hermanowicz, 2001). Since the biofilm itself consists of individual particles, bacteria, individual-based modeling (IBM) is a natural modeling choice. Mechanisms concerning a single cell, such as division and mobility, can be investigated in these models. Furthermore, the explicit nature of the time integration scheme allows for parallel solvers and domain decomposition, making this approach scalable as well as suitable for a large number of microbial agents. Several open-source software systems are available using this approach (see Lardon et al., 2011; Li et al., 2019; Naylor et al., 2017; Tack et al., 2017; Verhulst et al., 2011). Besides IBM, there are also continuum-based models describing not only the individual bacterium, but also the behavior of the biofilm as a whole. Here, the biofilm is homogenized, meaning that the material parameters are averaged over the whole inside of the biofilm, whether bacteria, EPS or voids are present. Rath et al. (2017) showed the precision of such an approach by combining experimental studies and numerical simulation. This second, continuum-based, approach is further investigated in this work.

In literature, there are two prominent approaches to modeling biological growth. The first one is commonly known and was developed by Rodriguez et al. (1994). The idea is to split the deformation tensor multiplicatively in an elastic and a growth part in a similar way as done in plasticity models. Thereby, the growth part of the deformation tensor describes an intermediate stress-free state. That way, changes in volume are included in the model, but no changes to the mass are present. Consequently, the density changes as a result of volume change. Additional constraint, like the constant density assumptions (see Soleimani, 2019), are necessary for the model to depict a change in mass, leading to a not-purely volumetric approach. Another way to include changes in mass into a model, the second approach, a density-based approach, can be used. This approach is used for example by Waffenschmidt et al. (2012). There, an energy-driven evolution equation is used to update the growing tissue by adding mass. Since the addition of mass in this approach is directly linked with the addition of the equivalent amount of volume, the density can never change in the density-based growth approach. In this paper, both approaches are unified in the context of a novel hybrid formalism derived from the stationarity principle. The interested reader is referred to Capriz and Mariano (2003) for a general theory on material modeling based on the stationarity principle. In the present work, an extension of the Hamilton principle is used. The extension of the Hamilton principle was first described by Junker and Balzani (2021) to provide a unifying theory for coupled problems and dissipative microstructure evolution and is extended even further in this work. The newly introduced framework offers a physically sound strategy to derive transient field equations for all state variables. Compared to classical approaches for deriving material models, like the principle of maximum dissipation, the principle of the minimum of the dissipation potential and others, the extended Hamilton principle provides direct access to transient field equations for all internal variables. Other main advantages are:

- since the extended Hamilton principle derives itself from the first and second law of thermodynamics, the thermodynamical consistency is automatically ensured. Existing phenomenological and heuristic model need to always check for thermodynamical consistency.
- coupling between variables will occur naturally without them being explicitly modeled. They may offer insights which might otherwise be hard to see and thus to investigate.
- a Hamilton-based approach directly yields holistic space-time formulations which are beneficial for a proper numerical treatment. For more information on space-time variational material modeling, the reader is referred to Junker and Wick (2024).

## Multi-physics mathematical model

In the upcoming chapter, the mathematical model is expounded. The presented model is based on two main assumptions. The first one is that growth and remodeling strongly impact both, the potentials in the biological body as well as the work along the process. The second assumption states that the energy necessary for growth is provided by nutrients. For the mathematical description, however, more assumptions are necessary, which are listed below.

- Only one species of bacteria is taken into account to prove the general concept of the modeling approach. The biofilm is furthermore homogenized. Voids and EPS are thus condensed in the continuous biofilm formulation.
- The growth formulation is based on the hyperbolic Monod equation (Monod, 1949).
- The mechanical contribution which emerges automatically from the Hamiltonian formulation is deemed to be small in comparison with other growth mechanisms and thus omitted from the model.
- Since biofilms consist of up to 98$\%$ water (Flemming & Wingender, 2001), incompressible material behavior can be assumed.
- Due to vastly different time scales of the diffusion process of the nutrients and the growth process, a steady state of already diffused nutrients is assumed.
- The effects of gravity on the biofilm are neglected for this work.

The basis of the mathematical description is the extended Hamilton principle which, as it is presented in Junker and Balzani (2021), applies to coupled problems and dissipative microstructure evolution. The dissipation, and therefore entropy production, in these models is always positive. It fulfills the first and second law of thermodynamics automatically. This fulfillment and consistency of the laws of thermodynamics are oftentimes not shown for biofilm growth models in the literature.

The extended Hamilton principle investigates the stationarity condition with regard to all (thermodynamic) state variables. Four state variables are chosen to model the growth—the first ones are the displacements $\textbf{u} = \textbf{u}(\textbf{x})$ in all spatial directions. To indicate the occurrence of biofilm in the discretized space, a biofilm state variable $\hat{\phi } = \hat{\phi }(x)$ is introduced. The biofilm state variable $\hat{\phi }$ is normalized by dividing by its maximum, i.e., $\phi = \frac{\hat{\phi }(x)}{\hat{\phi }_{\textrm{max}}}$. Hence, $\phi =1$ indicates a fully dense biofilm and $\phi =0$ indicates a locally void space without biomaterial. Intermediate states $\phi \in \;]0,1[$ are interpreted as partially dense biofilms. Thus, the variable $\phi$ can be interpreted as density. To indicate the presence of nutrients at a specific place, the nutrient concentration $c=c(\textbf{x})\in \;]0,1]$ is introduced as a third state variable. It is the normalized form of the amount of nutrients in a given volume, i.e., $c = \frac{\hat{c}(x)}{\hat{c}_{\textrm{max}}}$. Since the biofilm state variable $\phi$ and the nutrient concentration *c* are normalized, they are dimensionless. The fourth state variable used in this model is the local expansion parameter $\alpha$, which is also dimensionless. It describes the local expansion of the biofilm according to the volume-based approach.

### Kinematics of growth

**Fig. 1:**
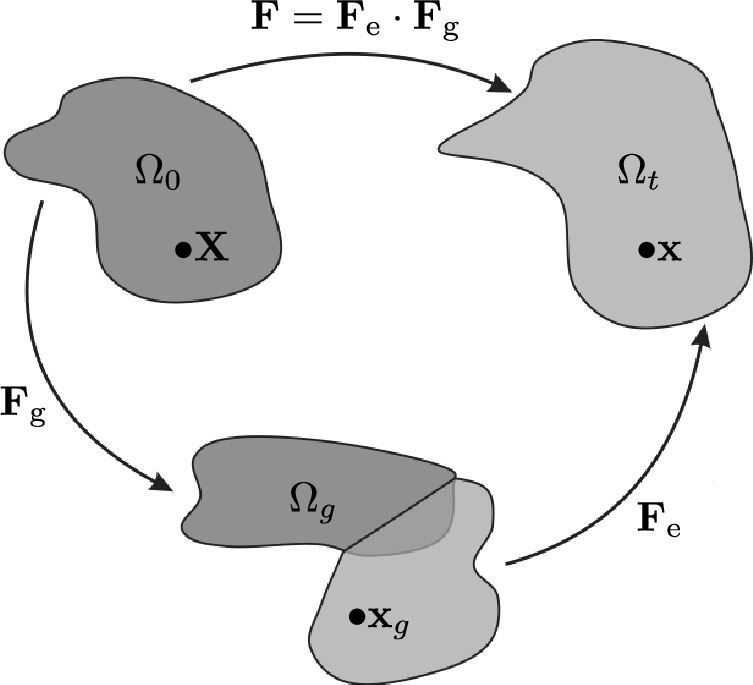
Visualization of the kinematic split after Soleimani (2017)

In this section, the kinematics of the model are explained. Similar to finite strain plasticity, the deformation gradient is split into a growth part and an elastic part such that $\textbf{F} = \textbf{F}_\textrm{e} \cdot \textbf{F}_\textrm{g}$. This kinematic split was first introduced into biomechanics by Rodriguez et al. (1994). It is exemplified in Fig. 1.

In further analogy to elasto-plasticity, a strain energy function is based on the elastic part. The growth part is constructed with the local expansion parameter $\alpha$ in the form of $\textbf{F}_\textrm{g} = \alpha \, \textbf{I}$, where $\textbf{I}$ is the identity tensor, i.e., isotropic growth is assumed. The local expansion parameter $\alpha$ itself is governed by an evolution equation which stems from the stationarity of the Hamilton functional $\mathcal {H}$ with respect to $\alpha$. The evolution equation of $\alpha$ connects the local expansion parameter $\alpha$ with the non-local biofilm state variable $\phi$. The derivation is shown in section 2.4. The growth part of the deformation gradient $\textbf{F}_\textrm{g}$ maps a point $\textbf{X}$ from the reference configuration $\Omega _0$ onto an intermediate, stress-free configuration $\Omega _g$. This introduced intermediate configuration may not be compatible since parts may overlap or holes may be created within this configuration. The elastic part of the deformation tensor $\textbf{F}_\textrm{e}$ then maps this intermediate configuration $\Omega _g$ onto the current configuration $\Omega _t$ to arrive at a physically sound, grown state. The elastic right Cauchy–Green deformation tensor as well as its isochoric counterpart can be expressed as

1 $$\begin{aligned} \textbf{C}_\textrm{e} = \textbf{F}_{\textrm{e}}^{\textrm{T}} \cdot \textbf{F}_{\textrm{e}} ,\qquad \tilde{\textbf{C}}_\textrm{e} = (\textrm{det} \, [ \textbf{F}_{\textrm{e}}])^{-2/3} \; \textbf{C}_{\textrm{e}}. \end{aligned}$$

### Extended Hamilton principle

The following chapter explains in detail the derivation of the extended Hamilton principle and the adjustments necessary to model biofilm growth. All equations are given in the reference configuration, if not explicitly stated otherwise. The first law of thermodynamics, i.e., the balance of energy, states that the rate of the total energy equals the power due to external loads.

2 $$\begin{aligned} \dot{\mathcal {E}} + \dot{\mathcal {K}} = \mathcal {P}^{\textrm{mech}} + \mathcal {P}^{\textrm{therm}} + \mathcal {P}^{\textrm{bio}} \end{aligned}$$

Here, $\dot{\mathcal {E}}$ is the change of the inner energy and $\dot{\mathcal {K}}$ is the change of the kinetic energy. On the right-hand side, the mechanical power $\mathcal {P}^{\textrm{mech}}$, the thermal power $\mathcal {P}^{\textrm{therm}}$ and the biological power $\mathcal {P}^{\textrm{bio}}$ change the total energy of the system $\mathcal {E} + \mathcal {K}$. The biological power $\mathcal {P}^{\textrm{bio}}$ is introduced to include all processes related to the change in energy performed by biological processes, such as growth and consumption. After integrating (2) over time, one obtains

3 $$\begin{aligned} \mathcal {E} + \mathcal {K} = \mathcal {W} + \mathcal {Q} + \mathcal {B} + \textrm{c}_{\textrm{int}} \end{aligned}$$

with the mechanical work $\mathcal {W}$, the thermal work $\mathcal {Q}$, the biological work $\mathcal {B}$ and some integration constant $\textrm{c}_\textrm{int}$. The integration constant is set to $\textrm{c}_\textrm{int} = 0$ since it only represents the energy of an arbitrary reference state. This reduces Equation (3) to

4 $$\begin{aligned} \mathcal {E} + \mathcal {K} = \mathcal {W} + \mathcal {Q} + \mathcal {B}. \end{aligned}$$

The inner energy consists of the total free energy as well as the heat. Integrated over the whole body $\Omega _0$ , it can be written as

5 $$\begin{aligned} \mathcal {E} = \int \limits _{{\Omega _0}} { \rho _0 \bar{\Psi }} \; \mathrm {d{V}} + \int \limits _{{\Omega _0}} \Theta \; \mathrm {{\rho _0 \bar{s}}} \; \mathrm {d{V}}, \end{aligned}$$

where ${\bar{\Psi }}$ is the mass specific free energy density which depends on the current physical state, e.g., the microstructure and deformation. The temperature is denoted by $\Theta$ and the mass specific entropy by $\bar{s}$. The initial density is referred to as $\rho _0$. The kinetic energy is given by

6 $$\begin{aligned} \mathcal {K} = \int \limits _{{\Omega _0}} \frac{1}{2} \rho_{0} \left\Vert \varvec{\dot{u}}\right\Vert ^2 \; \mathrm {d{V}}. \end{aligned}$$

The mechanical and thermal work are commonly described by

7 $$\begin{aligned}{} & {} \mathcal {W}=\int \limits _{{\Omega _0}} {\textbf {f}}^* \cdot {\textbf {u}} \; \mathrm {d{V}} + \int \limits _{\partial {\Omega _0}} {\textbf {t}}^* \cdot {\textbf {u}} \; \mathrm {d{A}}. \end{aligned}$$

8 $$\begin{aligned}{} & {} \mathcal {Q}=-\int \limits _{{\Omega _0}} {\int \limits _{t^\star }} \nabla _{\textbf{X}} \cdot {\textbf {q}}^* \; \textrm{d}{\bar{\textrm{t}}} \, \mathrm {d{V}} + \int \limits _{{\Omega _0}} {\int \limits _{t^\star }} \textrm{h}^{*} \; \textrm{d}{\bar{\textrm{t}}} \, \mathrm {d{V}}. \end{aligned}$$

The work performed by external traction forces ${\textbf {t}}^*$ and body forces ${\textbf {f}}^*$ as well as the heat flux vector ${\textbf {q}}^*$ and the heat source $\textrm{h}^*$ are marked with a star to indicate variables which are applied externally on the biofilm and have to be modeled to set the boundary value problem. Consequently, these quantities are assumed to be constant (dead loads) and thus unaffected during the calculation of variations. The biological work $\mathcal {B}$ is modeled analogously to the mechanical work performed on the body.

9 $$\begin{aligned} \mathcal {B} = \int \limits _{{\Omega _0}} \textrm{h}^{*}_{\phi } \; \phi \; \mathrm {d{V}} + \int \limits _{{\Omega _0}} \textrm{g}^{*}_{c} \; c \; \mathrm {d{V}}, \end{aligned}$$

where $\textrm{h}^{*}_{\phi }$ is the growth term, describing the growth of the biofilm and $\textrm{g}^{*}_{c}$ is the consumption term describing the consumption of nutrients. Similar to the forces ${\mathbf {f^*}}$ and ${\mathbf {t^*}}$ in the formulation of the mechanical work having an effect on the displacement $\textbf{u}$ of the body $\Omega _0$, the growth term $\textrm{h}^{*}_{\phi }$ and consumption term $\textrm{g}^{*}_{c}$ are having an effect on the biofilm state variable $\phi$ and the nutrients *c*. Both terms have to be modeled. The modeling approach presented in this work is stated in chapter 2.3.2. The second law of thermodynamics accounts for the irreversible flow of temperature in a non-universal direction.

10 $$\begin{aligned} \frac{\textrm{D}}{\textrm{Dt}} \int \limits _{{\Omega _0}} \rho _0 \bar{\textrm{s}} \; \textrm{dV} \ge 0 \end{aligned}$$

With the material time derivative in the reference configuration $\frac{\textrm{D}}{\textrm{Dt}} {:}{=}\frac{\partial }{\partial \textrm{t}}$. Two bodies which can exchange energy and are of different temperature will always compensate their thermal differences by increasing the temperature of the colder body while decreasing the temperature of the warmer body. This means, the heat energy, or entropy, in an adiabatic system can never decrease. The entropy can change in time due to entropy flux, an entropy source and entropy production $\Delta ^{\textrm{s}}$. We assume the entropy flux to be $\frac{\textbf{q}}{\Theta }$ and the entropy source to be $\frac{\textrm{h}^*}{\Theta }$ The entropy production $\Delta ^{\textrm{s}}$ accounts for the entropy production of dissipative processes related to growth. With that, the balance of entropy can be written after integration by parts as

11 $$\begin{aligned}&\int \limits _{{\Omega _0}} \Theta \, \rho _0 \bar{\textrm{s}} \, \mathrm {d{V}} -\int \limits _{{\Omega _0}} {\int \limits _{t^\star }} \nabla _{\textbf{X}} \cdot {\textbf {q}}^* \; \textrm{d}{\bar{\textrm{t}}} \, \mathrm {d{V}} + \int \limits _{{\Omega _0}} {\int \limits _{t^\star }} \textrm{h}^{*} \; \textrm{d}{\bar{\textrm{t}}} \, \mathrm {d{V}} =\nonumber \\ {}&- \int \limits _{{\Omega _0}} {\int \limits _{t^\star }} \dot{\Theta }\; \rho _0 \bar{\textrm{s}} \, \textrm{d}{\bar{\textrm{t}}}\, \mathrm {d{V}} - \int \limits _{{\Omega _0}} {\int \limits _{t^\star }} \frac{1}{\Theta } \; \textbf{q}^* \;\nabla _{\textbf{X}} \Theta \, \textrm{d}{\bar{\textrm{t}}} \, \mathrm {d{V}} \nonumber \\&- \int \limits _{{\Omega _0}} {\int \limits _{t^\star }} \Theta \Delta ^{\textrm{s}} \, \textrm{d}{\bar{\textrm{t}}} \, \mathrm {d{V}}, \end{aligned}$$

see Junker and Balzani (2021). Once energy is transformed into heat energy, it can only partly be transformed back into its original form of energy. The process of losing part of the energy in every process is called dissipation. The dissipated energy can be described as

12 $$\begin{aligned} \mathcal {D} = \int \limits _{{\Omega _0}} {\int \limits _{t^\star }} \Theta \Delta ^{\textrm{s}} \, \textrm{d}{\bar{\textrm{t}}} \, \mathrm {d{V}} {=}{:}\int \limits _{{\Omega _0}} \textrm{D}_{\textrm{diss}} \, \mathrm {d{V}} \end{aligned}$$

with the dissipated energy $\textrm{D}_{\textrm{diss}}$ which is modeled as $\textrm{D}_{\textrm{diss}} {:}{=}\textbf{p}^{\textrm{diss},*} \cdot \varvec{\xi }$. Here, $\varvec{\xi }$ denotes the vector of internal (thermodynamic) state variables needed to describe the physical system. The non-conservative forces are computed as $\textbf{p}^{\textrm{diss},*} {:}{=}\frac{\partial \Delta ^{\textrm{diss}}}{\partial \dot{\varvec{\xi }}}$ with the dissipation function $\Delta ^{\textrm{diss}}$, which needs to be modeled. In this work, a rate-dependent microstructure evolution is assumed which leads to the dissipative function modeled to be $\Delta ^{\textrm{diss}}=\frac{\eta }{2}\left\Vert \dot{\varvec{\xi }}\right\Vert ^2$. Further information for the modeling of the dissipative function can be found in Junker and Balzani (2021). The total potential is then introduced as

13 $$\begin{aligned} \mathcal {G} {:}{=}\int \limits _{{\Omega _0}} \rho _0 \bar{\Psi } \; \mathrm {d{V}} - \int \limits _{{\Omega _0}} {\textbf {f}}^* \cdot {\textbf {u}} \; \mathrm {d{V}} - \int \limits _{\partial {\Omega _0}} {\textbf {t}}^* \cdot {\textbf {u}} \; \mathrm {d{A}}. \end{aligned}$$

With the reformulations and the introduction of the second law of thermodynamics mentioned above, the balance of energies can be rewritten as

14 $$\begin{aligned} \mathcal {K} + \mathcal {G} = \mathcal {B} - \int \limits _{\Omega } {\int \limits _{t^\star }} \dot{\Theta }\; \rho _0 \bar{\textrm{s}} \, \textrm{d}{\bar{\textrm{t}}} \, \mathrm {d{V}} - \int \limits _{\Omega } {\int \limits _{t^\star }} \frac{1}{\Theta } \; \textbf{q}^* \;\nabla _{\textbf{X}} \Theta \, \textrm{d}{\bar{\textrm{t}}} \, \mathrm {d{V}} - \mathcal {D}. \end{aligned}$$

Since biofilms consist of up to 98$\%$ water (Flemming and Wingender 2001), incompressible material behavior can be assumed. This assumption leads to the condition that $\textrm{det} \, [\textbf{F}_{e}] = 1$, which is enforced by the constraint functional

15 $$\begin{aligned} \mathcal {C} = \int \limits _{{\Omega _0}} \, p \, (\textrm{det} \, [ \textbf{F}_{e}] - 1) \; \mathrm {d{V}} \end{aligned}$$

where *p* is the Lagrange parameter, which itself can be interpreted as hydrostatic stress. This assumption implies $\tilde{\textbf{C}}_\textrm{e} = \textbf{C}_\textrm{e}$ as can be seen from Eq. 1. The functional is necessarily zero, thus it can be added onto Eq. 14 which gives

16 $$\begin{aligned} \mathcal {K} + \mathcal {G} + \mathcal {C} = \mathcal {B} -\int \limits _{{\Omega _0}} {\int \limits _{t^\star }} \dot{\Theta }\; \bar{\textrm{s}} \, \textrm{d}{\bar{\textrm{t}}} \, \mathrm {d{V}} - \int \limits _{{\Omega _0}} {\int \limits _{t^\star }} \frac{1}{\Theta } \; \textbf{q}^* \;\nabla _{\textbf{X}} \Theta \, \textrm{d}{\bar{\textrm{t}}} \, \mathrm {d{V}}- \mathcal {D}. \end{aligned}$$

The energy balance can then be inserted into the action functional

17 $$\begin{aligned} \mathcal {A} {:}{=}\int _{\textbf{u}} \int _{{\Omega _0}} \rho \dot{\textbf{u}} \; \mathrm {d{V}} \,\textrm{d}\textbf{u} = \int _{\tau } \int _{{\Omega _0}} \rho \left\Vert \dot{\textbf{u}}\right\Vert ^{{2}} \; \mathrm {d{V}} \,\textrm{dt} = \int _\tau 2 \mathcal {K} \; \textrm{dt} \end{aligned}$$

with the time interval $\tau {:}{=}\{0, \textrm{T} \}$, where $\textrm{T}$ denotes the end time point. Inserting Eq. 3 in $\mathcal {A}$ results in the Hamilton functional

18 $$\begin{aligned} \mathcal {H} = \int _\tau (\mathcal {K} - \mathcal {G} - \mathcal {C} + \mathcal {B} - \mathcal {D}) \; \textrm{dt}. \end{aligned}$$

With the assumption of a quasi-static and isothermal process, i.e., $\mathcal {K} = 0$, $\dot{\Theta } = 0$ and $\nabla _{\textbf{X}} \Theta = 0$, $\mathcal {H}$ simplifies to

19 $$\begin{aligned} \mathcal {H} = \int \limits _{\tau }(\mathcal {G} + \mathcal {C} - \mathcal {B} + \mathcal {D})\; \textrm{dt} \rightarrow \underset{\textbf{u}, \varvec{\xi }}{\mathrm {stat.}} \end{aligned}$$

More details on the extended Hamilton principle including non-isothermal processes can be found in Junker and Balzani (2021) and Junker and Wick (2024).

### Material model

#### Energy density function

The material behavior has to be defined by an appropriate energy density function of the system. The energy chosen in this contribution is

20 $$\begin{aligned} {\Psi } = \phi ^{2} \; \frac{\mu }{2} \; (\textbf{I}: {\textbf{C}}_\textrm{e} - 3) + \frac{1}{2}\; \bar{\beta } \, \left\Vert \nabla _{{\textrm{X}}} \phi \right\Vert ^2 + \frac{1}{2}\; \bar{\textrm{d}} \, \left\Vert \nabla _{{\textrm{X}}} \textrm{c}\right\Vert ^2 - \bar{\textrm{k}}_{\alpha }\,\alpha \, \phi . \end{aligned}$$

The energy density ${\Psi }= \rho _0 \, \bar{\Psi }$ measured in $\frac{\textrm{J}}{\textrm{m}^3}$ consists of several different terms—the first one describes the mechanical behavior with the shear modulus $\mu$ in $\textrm{MPa}$ and the identity matrix $\textbf{I}$. Since the surrounding void is assumed to possess no mechanical stiffness, only the biofilm with the biofilm state variable $\phi$ is linked quadratically to this term. It is worth mentioning that the nonlinear dependence causes a non-convexity of the related condensed energy. In turn, localization of $\phi$ is intended. Both, the biofilm state variable $\phi$ and the nutrients field variable *c* have a diffusion term which regularizes the phase field with the parameters $\bar{\beta }$ and $\bar{\textrm{d}}$ , respectively. Both have Newton as their unit. The last term describes energy stored when local growth has evolved inside of the biofilm, i.e., $\alpha> 0,\; \phi > 0$, providing the linkage between the expansion parameter $\alpha$ and the non-local biofilm state variable $\phi$. It is scaled with the factor $\bar{\textrm{k}}_{\alpha }$. It is given in $\frac{\textrm{J}}{\textrm{m}^3}$.

#### Growth term $\mathrm {h^{*}_{\phi }}$ and consumption term $\mathrm {g^{*}_{c}}$

The second quantity which needs to be modeled is the growth term $\mathrm {h^{*}_{\phi }}$ in $\mathcal {B}$ in Eq. 9. It can be chosen to account for a variety of different growth approaches and possesses the unit $\frac{\textrm{J}}{\textrm{m}^3}$. We model it as

21 $$\begin{aligned} \mathrm {h^{*}_{\phi }} \; {:}{=} \; \tilde{r}(c) \; o(\nabla _{{\textrm{X}}} \phi , \nabla _{{\textrm{X}}} c). \end{aligned}$$

It consists of a growth function $\tilde{r}(c)$ and an orientation function $o(\nabla _{{\textrm{X}}} \phi , \nabla _{{\textrm{X}}} c)$. The growth function $\tilde{r}(c)$ is modeled with the Monod relation (see Monod (1949)) as

22 $$\begin{aligned} \tilde{r}(c) = \left\Vert \nabla _{{\textrm{X}}} \phi \right\Vert \, \frac{\bar{\textrm{r}} \, c}{\textrm{k} + c}, \end{aligned}$$

where $\bar{\textrm{r}}$ and $\textrm{k}$ are material constants. While $\bar{\textrm{r}}$ has the unit $\frac{\textrm{J}}{\textrm{m}^2}$, the material constant $\textrm{k}$ is dimensionless. The Monod equation is weighted with the norm of the gradient of the biofilm state variable $\phi$. The choice of including the norm of the gradient of $\phi$ promotes growth in the interface of biofilm and void. In turn, no non-local growth may happen in a region with homogeneous biofilm variable. As we will see later, local expansion, i.e., density-based growth, is still possible. The dimensionless orientation function indicates, as the name suggests, the orientation of maximal growth. In this work, an orientation function is chosen which promotes growth in the direction of the biggest nutrient gradient

23 $$\begin{aligned}{} & {} o(\nabla _{{\textrm{X}}} \phi , \nabla _{{\textrm{X}}} c) {:}{=}\textbf{n}_{\nabla \phi } \cdot \textbf{n}_{\nabla {c}} \quad \textrm{where} \quad \textbf{n}_{\nabla c} {:}{=}\frac{\nabla _{{\textrm{X}}} c}{\left\Vert \nabla _{{\textrm{X}}} c\right\Vert }\nonumber \\{} & {} \quad \textrm{and} \quad \textbf{n}_{\nabla \phi } {:}{=}\frac{\nabla _{{\textrm{X}}} \phi }{\left\Vert \nabla _{{\textrm{X}}} \phi \right\Vert } \end{aligned}$$

For the consumption, the simplest possible functional dependency, a linear relation, is chosen, i.e.,

24 $$\begin{aligned} \mathrm {g^{*}_{c}} = \bar{\textrm{g}} \, \phi \end{aligned}$$

where $\textrm{g}$ is the consumption factor with units of $\frac{\textrm{J}}{\textrm{m}^3}$. The higher the consumption factor $\textrm{g}$, the more consumption happens if biofilm is present.

### Evolution equations

After inserting the energy and the growth term into the extended Hamilton functional, its stationary condition with respect to the variables $\{ \textbf{u}, \varvec{\xi }\} = \{\textbf{u}, \phi , c,\alpha \}$

25 $$\begin{aligned} \delta \mathcal {H} = \delta _{\textbf{u}} \mathcal {H} + \delta _{\phi } \mathcal {H} + \delta _{c} \mathcal {H} + \delta _{\alpha } \mathcal {H} = 0 \qquad \forall \, \delta \textbf{u}, \, \delta \phi , \, \delta c, \, \delta \alpha \end{aligned}$$

needs to be evaluated. For the displacements, i.e., $\delta _{\textbf{u}} \mathcal {H} =0$, this leads to the principle of virtual work.

26 $$\begin{aligned} \delta _{\textbf{u}} \mathcal {H}&= \int \limits _{{\Omega _0}} \frac{\partial \Psi }{\partial \textbf{C}}_{\textrm{e}} : \delta \textbf{C}_{\textrm{e}} \; \mathrm {d{V}} - \int \limits _{{\Omega _0}} {\textbf {f}}^* \cdot \, \delta {\textbf {u}} \; \mathrm {d{V}} -\int \limits _{\partial {\Omega _0}} {\textbf {t}}^* \cdot \delta {\textbf {u}} \; \mathrm {d{A}}&\nonumber \\&\hspace{1.0em}+\int \limits _{{\Omega _0}} p \, \underbrace{\textrm{det}[\textbf{F}_{\textrm{e}}] \; \textbf{F}_{\textrm{e}}^{\mathrm {-T}}}_{=\, \textrm{cof}\,\textbf{F}_{\textrm{e}}} : \delta \textbf{F}_{\textrm{e}} \; \mathrm {d{V}} = 0 \qquad \forall \delta \textbf{u}&\end{aligned}$$

For the state growth variables $\phi$ and the concentration variable *c*, the stationarity conditions follow as

27 $$\begin{aligned} \delta _{\phi } \mathcal {H}&= \int \limits _{{\Omega _0}} \frac{\partial \Psi }{\partial \phi } \delta \phi \; \mathrm {d{V}} -\int \limits _{{\Omega _0}} \textrm{h}^{*}_{\phi } \; \delta \phi \; \mathrm {d{V}} \nonumber \\&+ \int \limits _{{\Omega _0}} \frac{\partial \Psi }{\partial \nabla _{{\textrm{X}}} \phi } \cdot \nabla _{{\textrm{X}}} \delta \phi \; \mathrm {d{V}} + \int \limits _{{\Omega _0}} \textrm{p}_{\phi }^{\textrm{diss},*} \delta \phi \; \mathrm {d{V}}&\nonumber \\&\overset{{\text {i.b.p.}}}{=} \int \limits _{{\Omega _0}} [\phi \mu \; (\textbf{I} : {\textbf{C}}_{\textrm{e}} - 3) + \textrm{h}_{\phi }^{*} {\, - \; \bar{\textrm{k}}_\alpha \alpha } - \bar{\beta } \, \nabla _{{\textrm{X}}}^{2} \phi + \eta _{\phi } \, \dot{\phi }\,] \delta \phi \; \mathrm {d{V}}&\nonumber \\&\hspace{1.0em}+ \int \limits _{\partial {\Omega _0}} \bar{\beta } \, \textbf{n} \cdot \nabla _{{\textrm{X}}} \phi \, \delta \phi \; \mathrm {d{A}} = 0 \qquad \forall \delta \phi&\end{aligned}$$

and

28 $$\begin{aligned} \delta _{\textrm{c}} \mathcal {H}&= \int \limits _{{\Omega _0}} \frac{\partial \Psi }{\partial c} \delta c \; \mathrm {d{V}} \nonumber \\&+ \int \limits _{{\Omega _0}} \frac{\partial \Psi }{\partial \nabla _{{\textrm{X}}} c} \cdot \nabla _{{\textrm{X}}} \delta c \; \mathrm {d{V}} + \int \limits _{{\Omega _0}} \textrm{p}_{c}^{\textrm{diss},*} \delta c \; \mathrm {d{V}} {\; + \int \limits _{\Omega _0} \mathrm {g^{*}_{c}} \phi \, \delta c \;\textrm{dV}}&\nonumber \\&\overset{{\text {i.b.p.}}}{=} \int \limits _{{\Omega _0}}[ \bar{\textrm{g}} \phi - \bar{\textrm{d}} \nabla _{{\textrm{X}}}^{2} c + \eta _{c} \dot{c}] \delta c \; \mathrm {d{V}} + \int \limits _{\partial {\Omega _0}} \bar{\textrm{d}} \textbf{n} \cdot \nabla _{{\textrm{X}}} c \, \delta c \; \mathrm {d{A}} = 0, \qquad \forall \delta c&\end{aligned}$$

respectively. The abbreviation “i.b.p.” is used to indicate where integration by parts was used.

#### Remark 1

The growth term $\mathrm {h_{\phi }^{*}}$ has a direct dependency on the variables $c, \nabla _{{\textrm{X}}} c, \nabla _{{\textrm{X}}}\phi$ i.e., $\mathrm {h_{\phi }^{*}} = \mathrm {h_{\phi }^{*}}(c, \nabla _{{\textrm{X}}} c, \nabla _{{\textrm{X}}}\phi )$. For the computation of the stationarity condition, this dependency is disregarded because the growth term $\mathrm {h_{\phi }^{*}}$ does not adjust itself to turn $\mathcal {H}$ stationary. It is imposed as a material property of the biofilm which needs to be modeled. In general, an arbitrary $\mathrm {h_{\phi }^{*}}$ can be chosen as long as it is not dependent on the rate of any internal variable. A suitable $\mathrm {h_{\phi }^{*}}$, however, consists only of parameters with a physical interpretation. Thus, we assume an unidirectional coupling between $\phi$ and *c*: the nutrient field variable *c* is not influenced by the biofilm state variable $\phi$. Equivalently, the same is true for the consumption $\mathrm {g_{c}^{*}} = \mathrm {g_{c}^{*}}(c)$.

The final part of the variation is

29 $$\begin{aligned}&\delta _{\alpha } \mathcal {H} = \int \limits _{{\Omega _0}} \biggl [\frac{\partial \Psi }{\partial \alpha } + \textrm{p}_{\alpha }^{\textrm{diss},*} \biggr ] \delta \alpha \; \mathrm {d{V}} = 0 \qquad \forall \delta \alpha . \end{aligned}$$

These stationarity conditions leave us with the local evolution equations for the biofilm state variable $\phi$, the nutrient concentration *c* and the local expansion parameter $\alpha$ as shown below.

30 $$\begin{aligned}&{\left\{ \begin{array}{ll} \eta _{\phi } \dot{\phi } - \bar{\beta } \nabla _{{\textrm{X}}}^2 \phi - \bar{\textrm{k}}_{\alpha } \alpha + \tilde{r}(c) \; o(\nabla _{{\textrm{X}}} \phi , \nabla _{{\textrm{X}}} c) \\\hspace{1.5cm} + \phi \mu \; (\textbf{I} : {\textbf{C}}_{\textrm{e}} - 3) = 0 \quad {\forall \textbf{X} \in \Omega _0} \\ \textbf{n} \cdot \nabla _{{\textrm{X}}} \phi = 0 \quad \forall \textbf{X} \in \partial \Omega _0 \end{array}\right. } \end{aligned}$$

31 $$\begin{aligned}&{\left\{ \begin{array}{ll} \eta _{c} \, \dot{c} - \bar{\textrm{d}} \, \nabla _{{\textrm{X}}}^2 c + \bar{\textrm{g}}\phi = 0 \quad {\forall \textbf{X} \in \Omega _0} \\ \textbf{n} \cdot \nabla _{{\textrm{X}}} c = 0 \quad \forall \textbf{X} \in \partial \Omega _0 \end{array}\right. }\end{aligned}$$

32 $$\begin{aligned}&{\left\{ \begin{array}{ll} \eta _{\alpha } \dot{\alpha } - \bar{\textrm{k}}_{\alpha } \phi \, = 0 \quad {\forall \textbf{X} \in \Omega _0} \end{array}\right. } \end{aligned}$$

In these equations, $\eta _{\phi }$, $\eta _{c}$ and $\eta _{\alpha }$ are the dynamic viscosity parameters with a unit of $\frac{\mathrm {k_g}}{\textrm{s}\,\textrm{m}}$. From Eq. 30, it can be seen that biofilm growth is coupled with the mechanical load. This link implies that growth is happening, where mechanical stress is present. Also mechanical stress and cell proliferation in biofilm is correlated, (see Chu et al. 2018). This link, however, is deemed to be very small in comparison with the growth due to other stimuli such as the concentration of nutrients surrounding the biofilm. In other words, the mechanical stiffness $\mu$ is assumed to be orders of magnitude smaller than every other parameter. Thus, mechanical influence on the growth in Eq. 30 is neglected, yielding

33 $$\begin{aligned} {\left\{ \begin{array}{ll} \eta _{\phi } \dot{\phi } - \bar{\beta } \nabla _{{\textrm{X}}}^2 \phi - \bar{\textrm{k}}_{\alpha } \alpha + \tilde{r}(c) \; o(\nabla _{{\textrm{X}}} \phi , \nabla _{{\textrm{X}}} c) &{}= 0 \quad \forall \textbf{X} \in \Omega _0 \\ \textbf{n} \cdot \nabla _{{\textrm{X}}} \phi &{}= 0 \quad \forall \textbf{X} \in \partial \Omega _0 \end{array}\right. } \end{aligned}$$

As mentioned above, the process of diffusion of nutrients is a very fast process in comparison with the growth of biofilms. This is why time homogenization is used. In mathematical terms, the viscosity parameter of the biofilm state variable and the expansion parameter is much larger than the viscosity parameter of the nutrients, i.e., $\eta _{\phi } \gg \eta _{c}$ and $\eta _{\alpha } \gg \eta _{c}$, i.e., a steady state for the concentration of nutrients. After dividing Eqs. 33, 31 and 32 by their respective viscosity parameter, the set of evolution equation emerges, which is used for calculation.

34 $$\begin{aligned}&{\left\{ \begin{array}{ll} \dot{\phi } - {\beta } \nabla _{{\textrm{X}}}^2 \phi - {\textrm{k}}_{\alpha } \alpha +\left\Vert \nabla _{{\textrm{X}}} \phi \right\Vert \, \frac{{\textrm{r}} \, c}{\textrm{k} + c}\; \textbf{n}_{\nabla \phi } \cdot \textbf{n}_{\nabla c} = 0 \quad {\forall \textbf{X} \in \Omega _0} \\ \textbf{n} \cdot \nabla _{{\textrm{X}}} \phi = 0 \quad \forall \textbf{X} \in \partial \Omega _0 \end{array}\right. }\end{aligned}$$

35 $$\begin{aligned}&{\left\{ \begin{array}{ll} \dot{c} - {\textrm{d}} \, \nabla _{{\textrm{X}}}^2 c + {\textrm{g}}\phi = 0 \quad {\forall \textbf{X} \in \Omega _0} \\ \textbf{n} \cdot \nabla _{{\textrm{X}}} c = 0 \quad \forall \textbf{X} \in \partial \Omega _0 \end{array}\right. }\end{aligned}$$

36 $$\begin{aligned}&{\left\{ \begin{array}{ll} \dot{\alpha } - {\textrm{k}}_{\alpha } \phi \, = 0 \quad {\forall \textbf{X} \in \Omega _0} \end{array}\right. } \end{aligned}$$

For the computations, we set $\eta _c = 10^{-10}$. Since we also set $\eta _\phi = \eta _c$, $\mathrm {k_\alpha }$ stays the same in Eq. 34 and in Eq. 36. Parameters which have been divided by their respective $\eta$ will lose their bar, i.e., $\bullet = \frac{\bar{\bullet }}{\eta }$.

## Numerical implementation

The adopted numerical method is based on the standard Galerkin FEM. The topology of the utilized element is a brick with eight nodes. There exist five unknowns per corner node, namely the displacements $\textbf{u} = (u,v,w)^\textrm{T}$, the concentrations of nutrients *c*, and the biofilm state variable $\phi$. The hydrostatic stress *p* is treated as a constant over the entire element. The chosen finite element formulation is equivalent to a standard H1P0 element in which the interpolation function for the rest of the field variables is assumed to be linear. A static condensation scheme is utilized to eliminate the hydrostatic stress at the element level. Additionally, the variable $\alpha$ which is defined as an internal local variable at the Gauss points is governed by the evolution equation 36 obtained from the Hamilton principle.

The weak form of the governing equations presented in Eqs. 26, 27 and 28 provide the basis for the numerical implementation. The implementation of the multi-field problem in hand was carried out using AceGen, see (Korelc and Wriggers 2016), which is a package in MATHEMATICA for automatic differentiation. Linearizing the weak forms gives the stiffness matrix that can be tailored to a user element in FORTRAN an implemented in any FEM solver. Here, ANSYS is used for this purpose. The objective of the numerical strategy is to find an algorithm for the update of field variables at the current time step, namely $u_{n+1},v_{n+1},w_{n+1},\textrm{c}_{n+1}$, and $\phi _{n+1}$. The subscript $n+1$ highlights the association of the variable with the current time step. Similarly, the previous values of the variables are denoted by *n* in the subscript. For the sake of conciseness and clarity, the numerical algorithm (pseudocode) is summarized in Table 1.

**Table 1:** Implementation algorithm in the AceGen

1. Interpolate the field variables $D_I$ (components of displacement, concentration, density) using standard linear FEM shape functions $N_I$
$\qquad D=\sum _{I}^{M} N_I{D}_I, \quad D_I:=u_I,v_I,w_I,{p},c_I,\phi _I$ $\quad M$:number of nodes
$$\qquad \mathbb {D}_e:=\bigcup _{I}^{N}D_I=\left( u_1,v_1,w_1,c_1,\phi _1,...,u_{N},v_{N},w_{N},c_{N},\phi _N,\right)$$
2. Initialize the global Newton–Raphson using
$$\qquad {\textbf{F}}_{e(n+1)}={\textbf{F}}_{n+1} \cdot {\textbf{F}}^{-1}_{g(n)}$$
$$\qquad {J}_{e(n+1)}=\textrm{det}({\textbf{F}}_{e(n+1)})$$
$$\qquad {{\tilde{\textbf{C}}}}_{e(n+1)}=J^{-\frac{2}{3}}_{e(n+1)}{\textbf{F}}^{\textrm{T}}_{e(n+1)} \cdot {\textbf{F}}_{e(n+1)}$$
Compute $\Psi _{n+1}$ using Eq. 20 and $\mathbb {D}_{e(n+1)}$ as well as $\alpha _n$
$$\qquad {\varvec{\sigma }}_{n+1} =\frac{1}{{J}_{e(n+1)}}\frac{\partial {\Psi _{n+1}} }{\partial \textbf{F}_{e(n+1)}}\textbf{F}_{e(n+1)}^{\textrm{T}}$$
3. Local equation (at Gauss point): ${\mathbb {R}}_{\alpha }=0$
a. Solve the local problems, namely Eq. 36, at Gauss points to find the internal variables $\alpha _{n+1}$
$$\qquad {\mathbb {R}}_{\alpha }=\frac{(\alpha _{n+1}-\alpha _{n})}{(1+\alpha _{n+1})\Delta t} -\frac{\mathrm {{k_\alpha }}}{\eta _{\alpha }} \frac{(\phi _{n+1}-\phi _{n})}{\Delta t}$$
b. Update $${J}_{e(n+1)}$$, $${\textbf{F}}_{g(n+1)}$$ and $${\textbf{F}}_{e(n+1)}$$
c. Compute $\varvec{A}=\frac{\partial \alpha _{n+1}}{\partial \mathbb {D}_e}$ which is later needed for the tangent computation (step 5)
4. Compute $\delta \mathcal {H}$ using Eq. 25
$$\quad \delta \mathcal {H}=\int _{\Omega } [\textbf{S}_{e(n+1)}:\delta \textbf{C}_{e(n+1)}+({J}_{e(n+1)}-1)\delta p_{n+1}]\ \textrm{dv}$$
$$\qquad +\int _{\Omega } [\textrm{d} \, \nabla c_{n+1} \cdot \nabla \delta c_{n+1} +g \, \delta c_{n+1}]\ \textrm{dv}$$
$$\qquad +\int _{\Omega } [\beta \, \nabla \phi _{n+1} \cdot \nabla \delta \phi _{n+1}+\eta _{\phi }\frac{\phi _{n+1}^t-\phi _{n+1}^{t-1}}{\Delta t} \, \delta \phi _{n+1} -\textrm{r} \frac{ c_{n+1}}{\textrm{k}+c_{n+1}}\frac{\nabla \phi _{n+1}\cdot \nabla c_n}{|\nabla c_n|}\,\delta \phi _{n+1}]\ \textrm{dv}$$
5. Compute the residuum vector of the element ${\varvec{\mathbb {R}}}_{e}=\frac{\partial \delta \mathcal {H}}{\partial {\delta \mathbb {D}}_e}$
6. Compute the element stiffness matrix taking into account the local internal variables
$${\varvec{\mathbb {K}}}_{e}={\frac{\partial \varvec{\mathbb {R}}_e}{\partial {\mathbb {D}}_e}} |_{\frac{\partial \alpha }{\partial {\mathbb {D}}_e}=\varvec{A}}$$
7. Do the static condensation (Schur complement) for the pressure degree of freedom (*p*) in the element stiffness matrix $\mathbb {K}_{e}$ which has been expressed in terms of submatrixes $K_{DD}, K_{Dp},K_{pD}$ and $K_{pp}$
$$\qquad \mathbb {K}_{e}=\begin{bmatrix} K_{DD} &{} K_{Dp} \\ K_{pD} &{} K_{pp} \\ \end{bmatrix}_{41\times 41} \xrightarrow []{\text {Schur \ complement}}\mathbb {K}_e= [K_{DD}- K_{Dp}K^{-1}_{pp}K_{pD}]_{40 \times 40}$$
8. Global Newton–Raphson iteration: $(\mathbb {R},\mathbb {D},\mathbb {K})=\bigcup ^{\mathrm {all\ elements}}( \mathbb {R}_e,\mathbb {D}_e,\mathbb {K}_e)$
DO WHILE $\left\| \varvec{\mathbb {R}} \right\| \ge \textrm{tol}$ (check the global convergence)
Repeat steps (2) to (6)
$$\mathbb {D} \Leftarrow \mathbb {D}+\Delta \mathbb {D}, \quad \Delta \mathbb {D}=-\mathbb {K}^{-1} \mathbb {R}$$
END DO
9. Go to the next time step and start from step (2)

## Numerical examples

In the following chapter, selected results of numerical simulations of the model will be presented to verify the validity of the model. For the numerical examples, parameters have been chosen that provide a good phenomenological match to biofilm growth. These parameters are listed in Table 2. If a simulation, presented in the following, deviates from these values, it is explicitly stated. The time step in all simulations was defined by substeps. The nominal amount of substeps was $10^3$, if the simulation did not converge; however, bisection occurred automatically up to a maximum amount of substeps of $10^6$. Biofilm formation time can vary widely based on nutrient availability or species of microorganisms involved. To account for differences in time, a normalized time $\textrm{T}^* = \frac{t}{t_\textrm{ref}}$, with the actual time *t* and a reference time $t_{\textrm{ref}}$ which is chosen based on the conditions for growth at hand such that the normalized time $\textrm{T}^* \in [0,1]$.

**Table 2:** Parameter used in subsequent simulations. The shear modulus $\mu$ is equivalent to a Young’s modulus $E=10 \; \textrm{Pa}$ and Poisson ratio $\nu = 0.49$ (Soleimani, 2019)

Parameter		Value	Units
Shear modulus	$\mu$	3.3557	Pa
nutrient diffusivity	d	$10^{10}$	$\mu \textrm{m}^{2} \cdot {\textrm{T}^{*-1}}$
Phase-field regularization parameter	$\beta$	2	$\mu \textrm{m}^{2} \cdot {\textrm{T}^{*-1}}$
Growth factor	$\mathrm {k_{\alpha }}$	$10^{-3}$	${\textrm{T}^{*-1}}$
Half-velocity constant	$\textrm{k}$	1	-
Consumption parameter	$\textrm{g}$	$10^8$	${\textrm{T}^{*-1}}$
Growth parameter	$\textrm{r}$	100	${\textrm{T}^{*-1}}$

### Test case 1: directional growth

For the first boundary value problem, a cube with the dimensions of $20\times 20\times 20\mu$m is discretized. The dimensions of the mesh are 1$\mu$m in each direction.

**Fig. 2:**
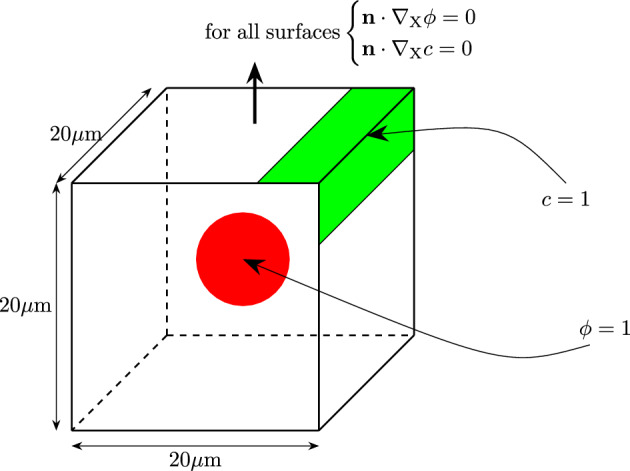
Schematic figure of the boundary conditions. The position of the nodes where $\phi =1$ is colored red whereas the position of nodes where $c=1$ is colored green

The biofilm state variable $\phi$ is applied as a constant value of $\phi = 1$ to all nodes within a radius of 5$\mu$m at the center of the cube. In one of the corners, the value of the nutrients is fixed to be $c = 1$ at all times to provide the nutrients necessary for growth. These boundary conditions are visualized in Fig. 2.

**Fig. 3:**
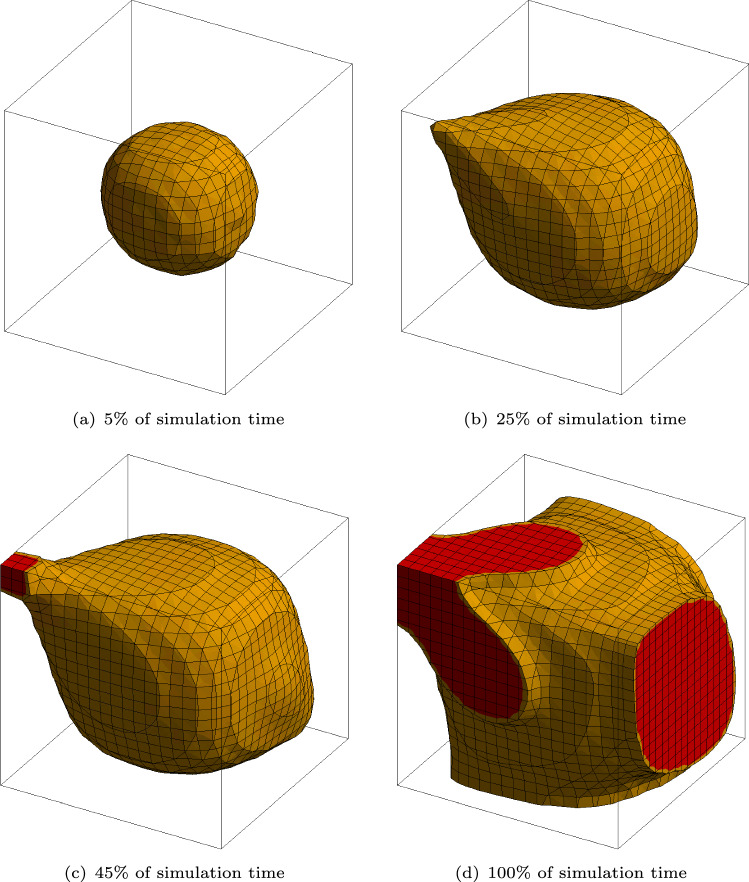
Simulation results at different times of the first test case. The isosurface, where the biofilm state variable is $\phi \ge 0.8$, is shown. The biofilm is growing toward the top left corner where the nutrient variable is set constant to $c=1$

Figure 3 shows a 3D rendering of all the values of $\phi \ge 0.8$ at different times of the simulation. It can clearly be seen that the biofilm state variable $\phi$ evolves toward the corner with nutrient source. Additionally, the volume of the center sphere increases. The development of three variables, the biofilm state variable $\phi$, the nutrient concentration *c* and the hydrostatic stress *p*, is depicted in Table 3. The pictures in the table show the same diagonal cut through the cubic domain. The cut is performed diagonally through the middle of the domain in such a way that the corner with the applied nutrients is at the top right. In the first column, the biofilm state variable $\phi$ is plotted. As in Fig. 3, the growth toward the nutrients can be seen clearly. This directional growth stems from the formulation of $\textrm{h}_{\phi }^* = \textrm{r}\, \frac{{c}}{\textrm{k} + {c}} \nabla _{{\textrm{X}}}\phi \cdot \textbf{n}_{\nabla {c}}$ which contains the product of gradients of the nutrient concentration *c* and of the biofilm state variable $\phi$. Thus, growth in the direction of the steepest gradient of nutrient concentration is enhanced which is supported by experimental observations in Hornung et al. (2018). In the simulation results, there is another observation which can be traced back to the formulation of $\textrm{h}_{\phi }^*$

**Table 3:**
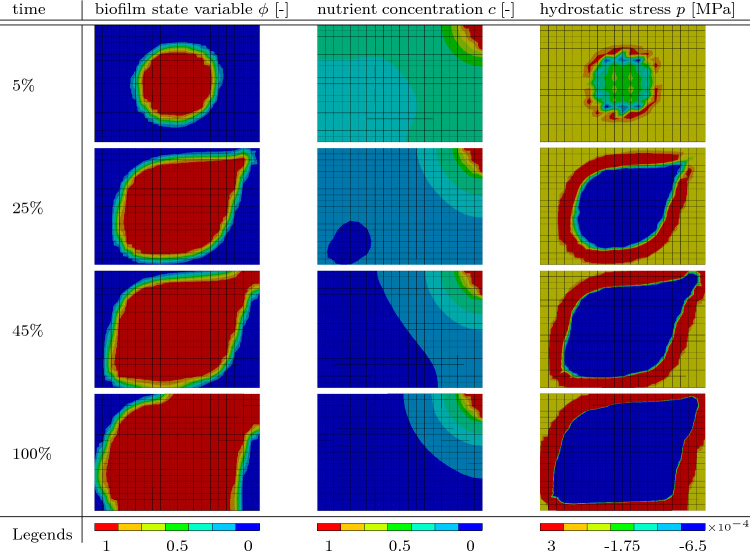
Variable distribution in the middle cross section of the first numerical example presented

**Fig. 4:**
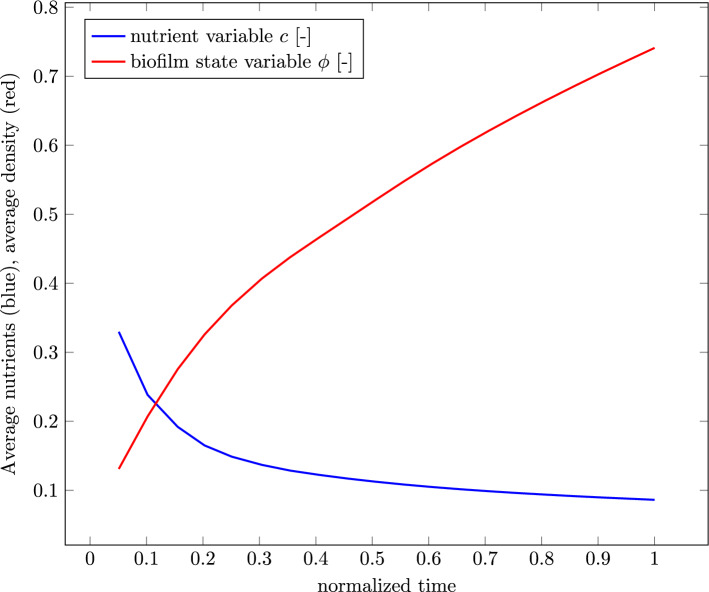
Average value of the nutrient concentration *c* (blue) and the biofilm state variable $\phi$ (red) throughout the whole discretized domain

Since there is also a gradient of the nutrients in the opposite side of the biofilm, i.e., toward the corner, opposite to where the nutrient concentration is set to $c=1$, the biofilm growth is also enhanced in this direction resulting in a slight egg-like shape. The egg-like shape can also be observed in Fig. 3. There is less growth on the sides of the biofilm facing the other corners of the cube because of the nature of the vector product between the two gradients. At these sides, the gradient of biofilm and the gradient of nutrients are almost perpendicular to each other resulting in a small value for the vector product and consequently in minimal to no growth. Regardless, an overall increase in the spheres volume can be observed, as mentioned before. In the second column of Table 3, the nutrients are portrayed. Since the diffusion is a fast process in relation to growth, the nutrients are already diffused through the whole domain at the start of the simulation at $5\%$ of the simulation time. While the growth of the biofilm takes place, the nutrients are getting depleted. Depletion first takes place in the section of the biofilm furthest from the source of nutrients which results ultimately in the already complex interactions between the two gradient vectors. Since new nutrients are always diffusing from the top corner, the amount of total nutrients in the domain will never reach zero but rather an equilibrium state where as much nutrients are getting depleted as can diffuse through the domain. The equilibrium state is being approached by the termination of the simulation, which is illustrated in the graph depicted in Fig. 4.

The graph shows the average nutrients throughout the whole domain in blue and the average biofilm state variable in the whole domain in red. The nutrients tend to an equilibrium state at an average nutrients value of around $10\%$. In accordance to the Monod relation used in the formulation of $\textrm{h}_{\phi }^*$, the biofilm state variable first grows rapidly before the growth rate decelerates and also tends to an equilibrium state. In this case, the equilibrium is not achieved and the whole domain would fill up with biofilm if continued. In the third column in Table 3, the hydrostatic stress *p* can be seen. Inside the biofilm, hydrostatic pressure can be observed. On the outer edge of the biofilm is a ring of tension. Outside of the biofilm, the pressure is zero. The stress is a direct cause of correlating the biofilm state variable $\phi$ with the deformation tensor $\textbf{F}$ through the definition of the growth part of the deformation tensor as $\textbf{F}_\textrm{g} = \alpha \textbf{I}$.

### Biofilm on nutrients

**Fig. 5:**
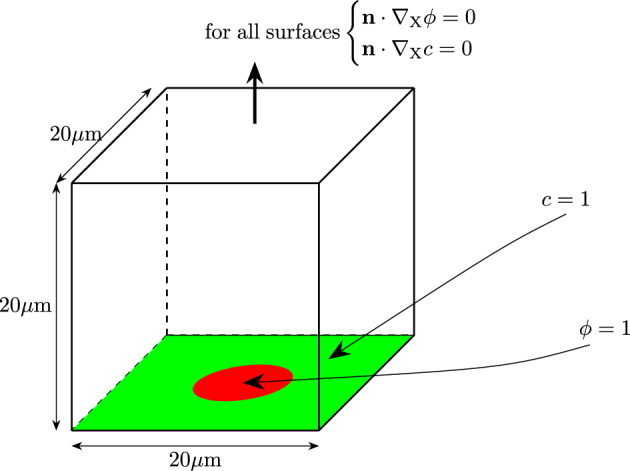
Schematic figure of the boundary conditions. The position of the nodes where $\phi =1$ is colored red and $c=1$ is colored green

To model the growth behavior of biofilm sitting on nutrients, different boundary values are chosen which can be seen in Fig. 5. The nodes at the bottom of the discretized $20\times 20\times 20\mu$m domain are set to a nutrient concentration of $c=1$. In the plane directly above, the initial biofilm is placed in a centered circle with a diameter of 5$\mu$m by setting the value for the biofilm state variable to $\phi =1$.

**Fig. 6:**
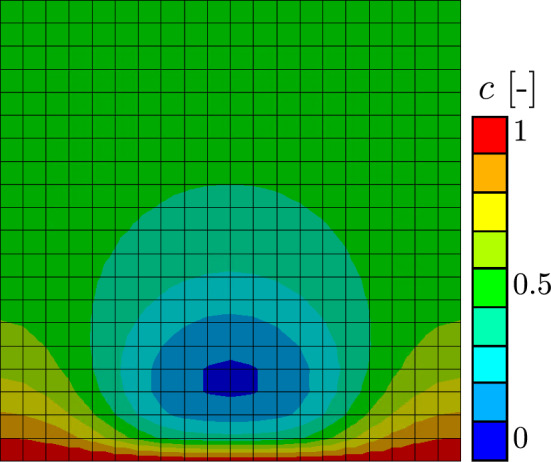
General behavior of the nutrient field from the agar simulations. The blue area on top of the biofilm indicates low nutrient level, the red area at the bottom a nutrient level of $c=1$. This particular nutrients field is taken from the “low” agar simulation at the start of the simulation

**Fig. 7:**
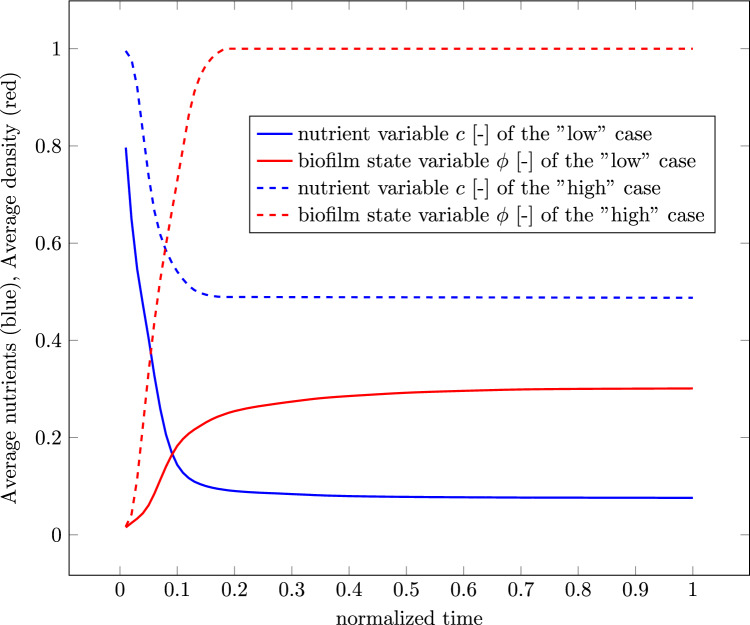
Average values of the nutrient variable *c* in blue and biofilm state variable $\phi$ in red over the simulation time. The “high” case is illustrated as a dashed line, the “low” case in a solid line

This setup is subdivided into two sub-cases with different consumption parameters $\textrm{g}$. Simulation results with $\textrm{g}=10^{10}\, {\textrm{T}^{*-1}}$ will be labeled as “low,” and simulation results with a consumption parameter of $\textrm{g}=10^8 \,{\textrm{T}^{*-1}}$ will be labeled as “high.” All other parameters are equal to the parameters presented in Table 2. Table 4 shows the distribution of the biofilm state variable $\phi$ for both sub-cases. Purely as a result of using different material parameters, two vastly different growing pattern can be seen. Right from the start, the results from the simulation with the lower consumption factor $\textrm{g}$ grows much higher and filling the entire discretized cube after just $13\%$ of simulation time. During the simulation, first a mushroom-shaped structure and then a droplet-like structure can be observed, which is in accordance to observations in *in vitro* experiments with biofilm-forming bacteria such as Toyofuku et al. (2016). In contrast, the simulation with a higher consumption factor grows only to a certain height, after which the biofilm fills out the edges of the discretized cube, forming a biofilm layer at the bottom. This vastly different behavior can be explained by the different nutrient fields emerging from different consumption factors. The general nutrient field emerging from these kinds of boundary value problem is shown in Fig. 6. Above the biofilm is a zone where there are little to no nutrients. This area is dark blue in the illustration. On the sides of the biofilm, the gradient of nutrient concentration is less steep than directly in the middle where there is biofilm which depletes the nutrients diffusing from the bottom of the cube. With a higher consumption factor, there are no nutrients on top of the biofilm to grow into. A small consumption factor allows the nutrients to be available in a vast quantity above the biofilm. The graph in Fig. 7 shows both, the average nutrients concentration *c* in blue and the average value of the biofilm state variable *ϕ* in red of both sub-cases over the whole domain.﻿

**Table 4:**
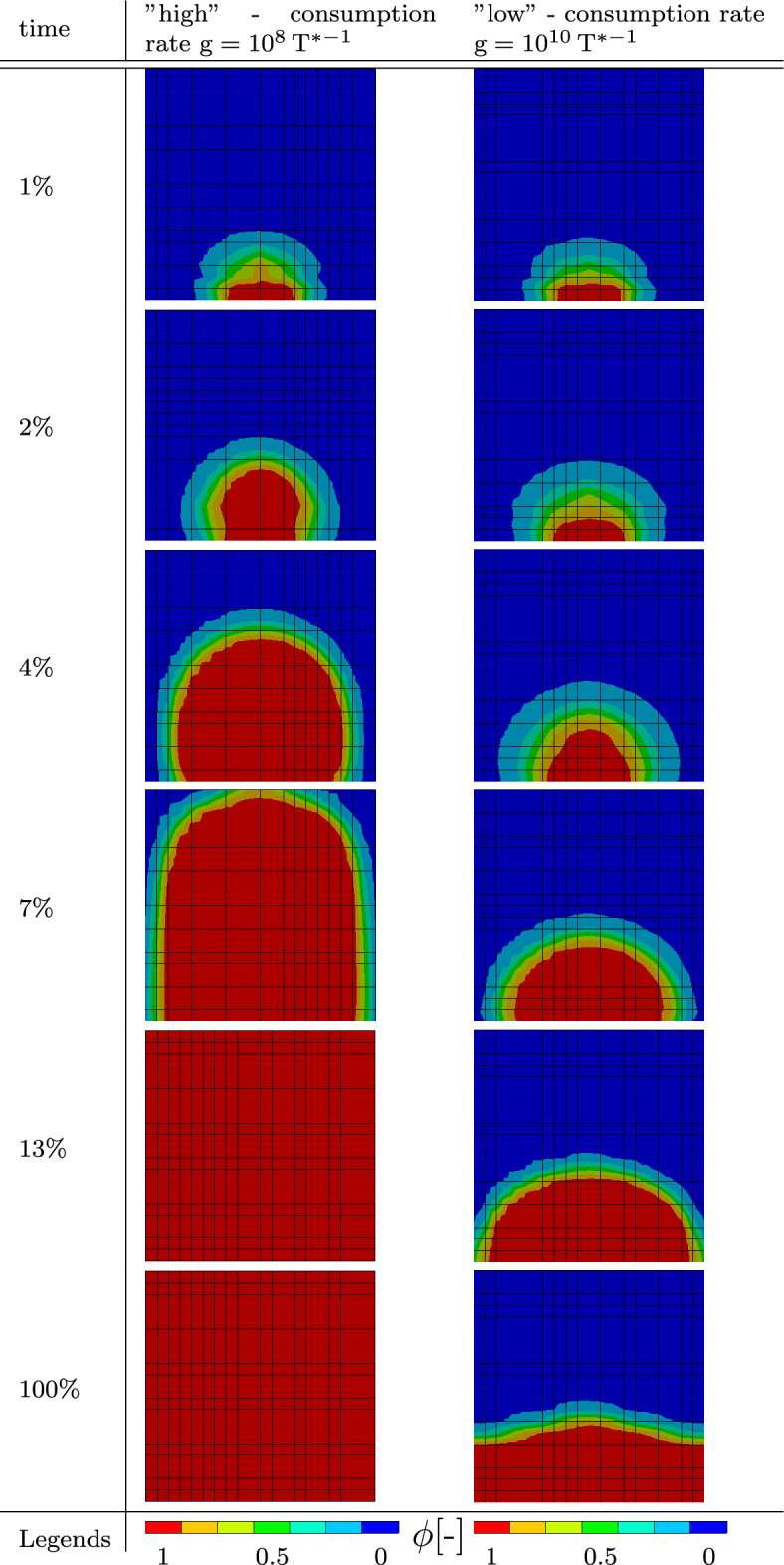
Distribution of the state growth variable $\phi$ of the model resembling an agar plate at different times throughout the “high” and “low” boundary value problem

**Fig. 8:**
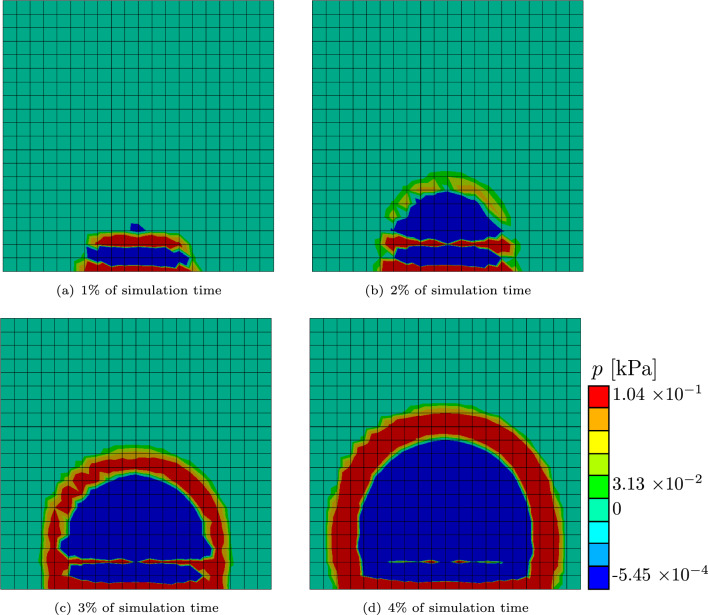
Hydrostatic stress distribution in the biofilm with the agar simulations. Here, it is shown exemplary with four results from the “high” agar simulation at different times. The teal color indicates a hydrostatic stress of $p{\text{ = 0}}$, red indicates tension and blue compression. The absolute values are dependent of Young’s modulus which needs to be adjusted to fit real biofilm in future works

The “high” case is illustrated by the dashed line, the “low” case by a solid one. The general behavior of both sub-cases is very similar. It can be seen that in both cases, an equilibrium state has been reached and neither the nutrient concentration nor the biofilm state variable changes after that state has been reached. It also shows that despite the fact that the “high” sub-case fills up the discretized domain after $13\%$ of simulation time, the nutrient concentrations equilibrium is significantly higher than the nutrient concentration from the “low” sub-case. The hydrostatic stress distribution inside both simulation shows a similar pattern as well. In Fig. 8, qualitative results of the “high” case are shown. The following observation holds true for both cases though: the blue region is under pressure, the red and orange regions are under tension, and the region colored in green is zero. At the very start of the simulation, in Fig. 8a, tension can be seen in the regions where the biofilm first grows toward the bottom of the discretized domain and toward the nutrients at the top of the biofilm. Inside the biofilm, there is pressure at all times of the simulation. Once it grows to the top, a new region of tension forms, as can be seen in Fig. 8b–d. The initial region of tension inside the biofilm vanishes slowly.

### Growth against rigid obstacles

**Fig. 9:**
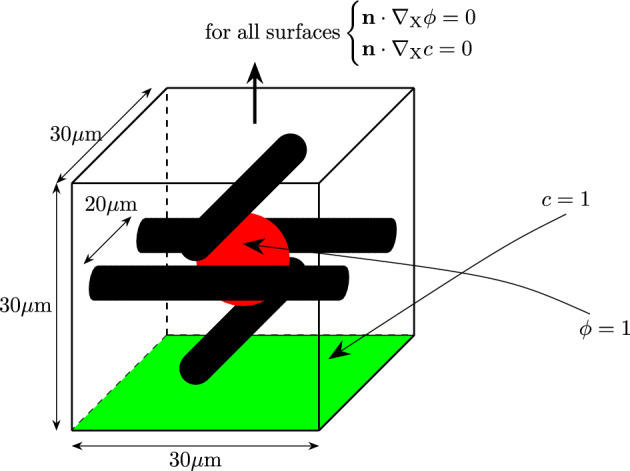
Schematic figure of the boundary conditions. The position of the nodes where $\phi =1$ is colored red, where $c=1$ is colored green and the rigid obstacles are black

The geometry of the obstacle test case is taken from Albero et al. (2014) and was later used by Soleimani (2019) and Soleimani et al. (2020). It is schematically visualized in Fig. 9.

**Fig. 10:**
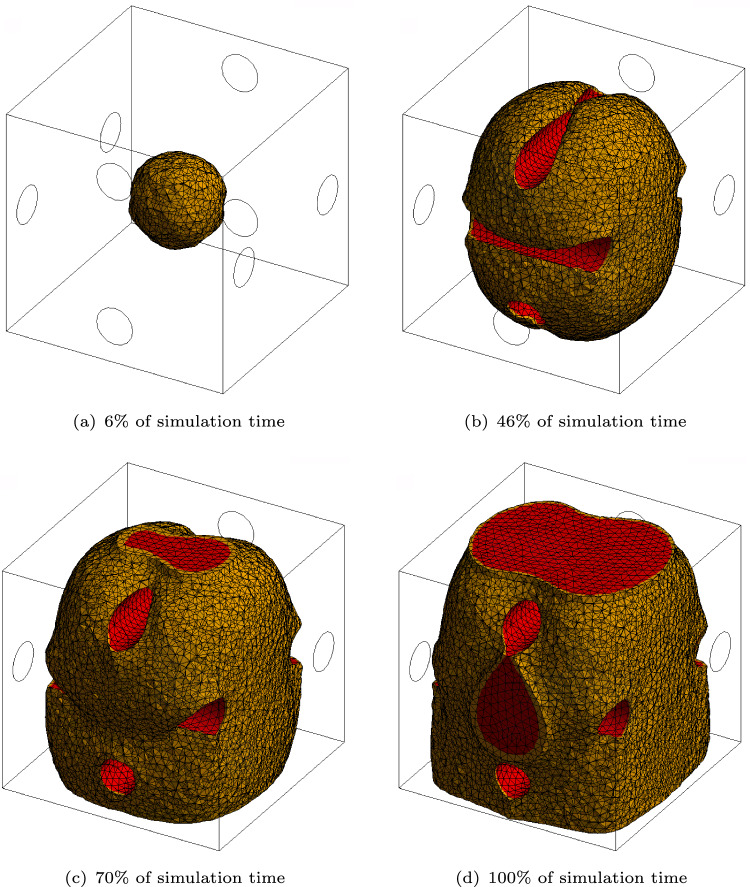
Three-dimensional rendering of the biofilm state variable $\phi \ge 0.8$ at different times throughout the simulation. The growth around rigid obstacles can be observed

The idea is to provide a highly complex environment for the biofilm to grow in, containing regions posing constraints to the growth process. It consists of a $30\times 30\times 30\mu$m cube with four columns. The columns act as rigid obstacles for the biofilm growth. They are modeled as solid objects where all degrees of freedom are set to zero. The contact between the solid bars and the cube containing biofilm is defined as frictionless. The domain in which the biofilm can develop can thus slide past the rigid tubes without penetrating them. The biofilm state variable is set to $\phi = 1$ in a sphere with a radius of 5$\mu$m located in the center of the cube. Similarly to the agar test case, the nodes of one side of the cube are set to a value of the nutrient concentration of $c=1$. Figure 10a–d shows a 3D rendering of all values of $\phi \ge 0.8$ at different times throughout the simulation. The sphere, in which the initial biofilm starts, can be seen in Fig. 10a. The biofilm then quickly grows around the rigid columns and reconnects. While self-contact can be an issue if new elements are created, the formulation presented here avoids this numerical challenge since the biofilm state variable is a field variable evolving inside the pre-discretized cube.

Table 5 shows a diagonal cut through the domain. The rigid columns can be seen as blue ovals since the cut is performed at a $45^\circ$ angle to both of them. The state growth variable $\phi$ and the nutrients concentration *c* are presented at the same simulation times as in Fig. 10. Not surprisingly, the nutrient concentration field shows a similar behavior as in the cases presented before.

**Table 5:**
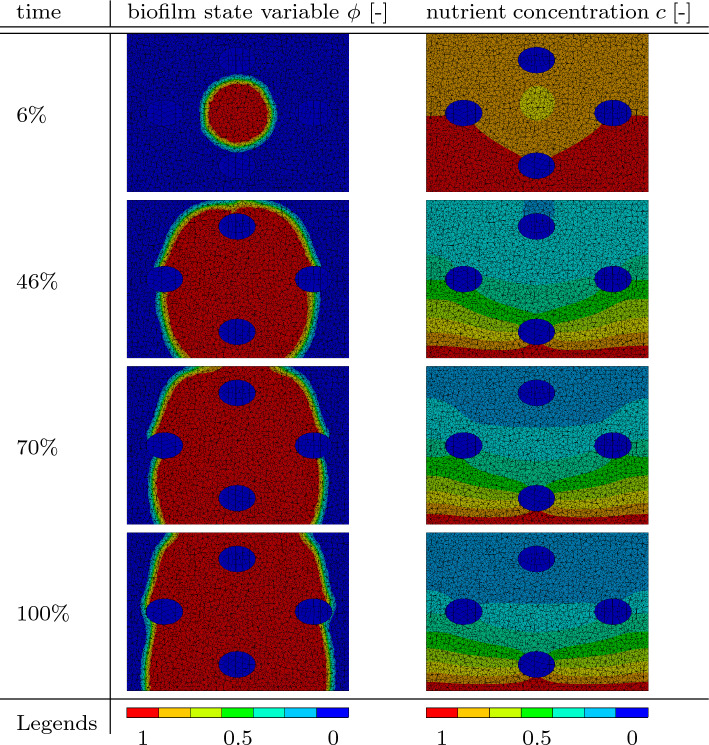
Results of the simulation containing obstacles. In the left column, the biofilm state variable $\phi$ is shown. In the right column, the nutrient concentration *c* is presented at different times throughout the simulation

### Maze

To show the versatility of the model, a complex maze geometry was designed. The model is semi-2D, i.e., the thickness of the model is one element and thus no three-dimensional effects can be captured. The maze has a diameter of $560 \mu \textrm{m}$ and thus is larger than the other boundary value problems presented. Therefore, the simulation parameters have been adjusted. The modified parameters are listed in Table 6.

**Table 6:** Parameter used in the maze simulation (all other parameters stay the same)

Parameter		Value	Units
Nutrient diffusivity	d	$10^{13}$	$\mu \textrm{m}^2 \cdot {\textrm{T}^{*-1}}$
Phase-field regularization parameter	$\beta$	10	$\mu \textrm{m}^2 \cdot {\textrm{T}^{*-1}}$
Growth factor	$\mathrm {k_{\alpha }}$	$10^{-6}$	${\textrm{T}^{*-1}}$
Growth parameter	$\textrm{r}$	200	${\textrm{T}^{*-1}}$

**Fig. 11:**
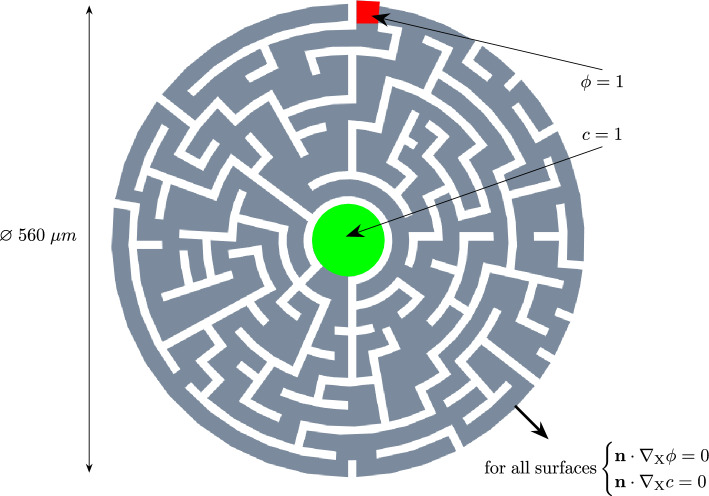
Geometry of the maze. The thickness is 4 $\mu m$. The undiscretized wall is 10 $\mu m$ wide

The geometry is depicted in Fig. 11. The maze itself consists of two bodies: the path where the biofilm and nutrients behave like in the boundary value problems shown prior and the walls where neither of them is present.

In addition to that, the wall is fixed in all three spatial directions. Between the two bodies, frictionless contact is modeled to allow the sliding between the path and the wall. The nodes in the circular area in the center of the maze are set to have a nutrient concentration of $c=1$. They diffuse through the maze following the path from the middle to the entrance of the maze where the initial biofilm colony, i.e., the nodes with $\phi =1$ are located at the start of the simulation. The diffusion process and subsequent depletion are shown in the right column of Table 7. The biofilm first grows from the entrance in all directions, since there are enough nutrients to allow growth, even if the biofilm grows into a dead-end. The bigger the dead-end is, the more nutrients diffused into it and the more biofilm grows into the dead-end. Once nutrients are depleted in the dead-end, the biofilm either stays at the level that was before, or it retracts. The retraction can be observed in the table at $40\%$ of the simulation time when compared to the following images. The points of interest are enhanced in Table 7. At the end of the simulation, the biofilm arrives at a steady state while also reaching the middle of the maze.

**Table 7:**
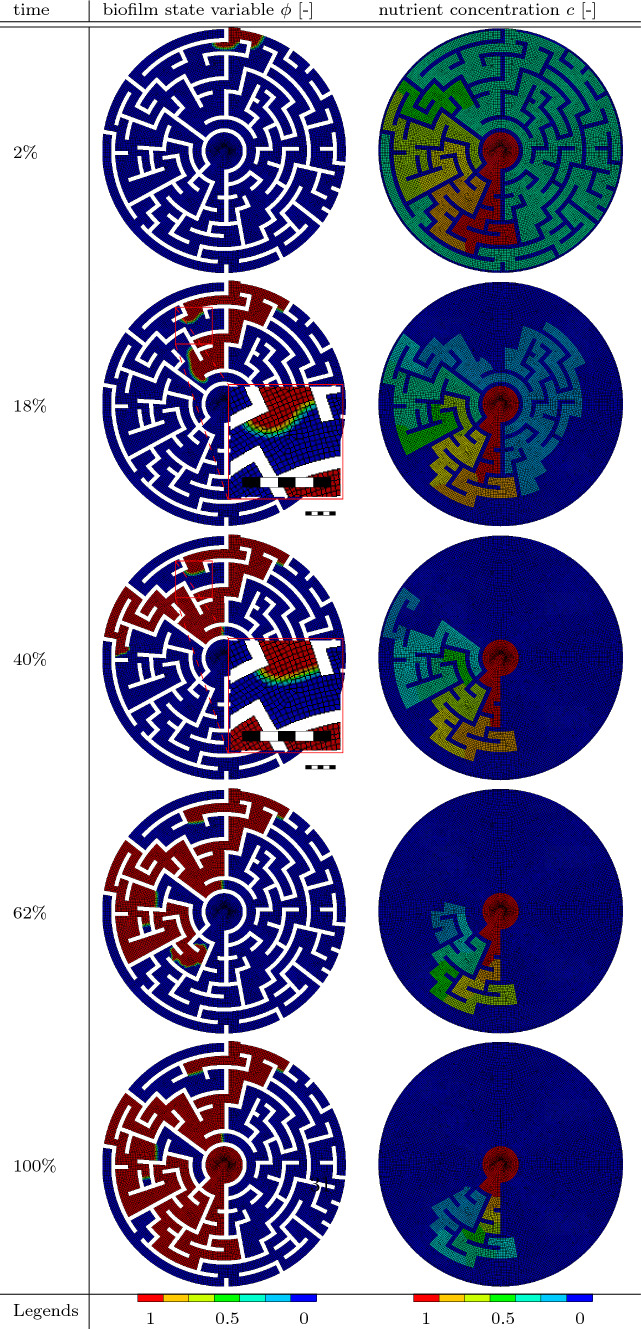
Results of the maze simulation. In the left column, the biofilm state variable $\phi$ is shown. In the right column, the nutrient concentration *c* is presented at different times throughout the simulation

### Grate

As a last example, another semi-2D boundary value problem is presented.

**Fig. 12:**
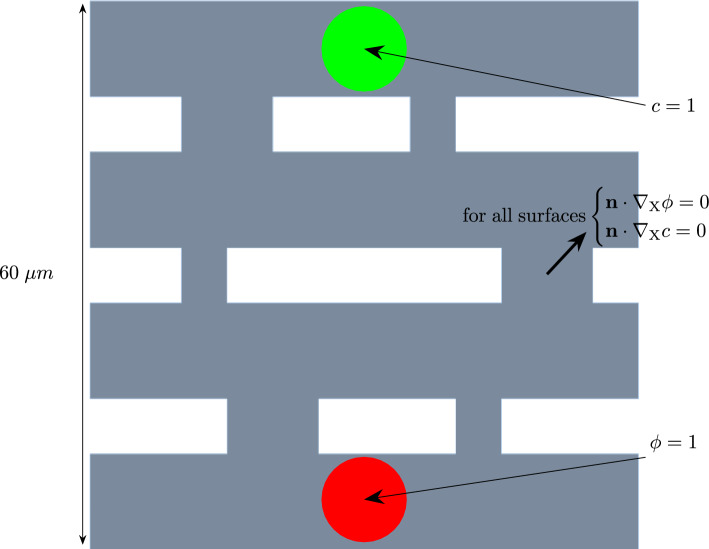
Geometry of the grate. The thickness is 1 $\mu m$

**Table 8:**
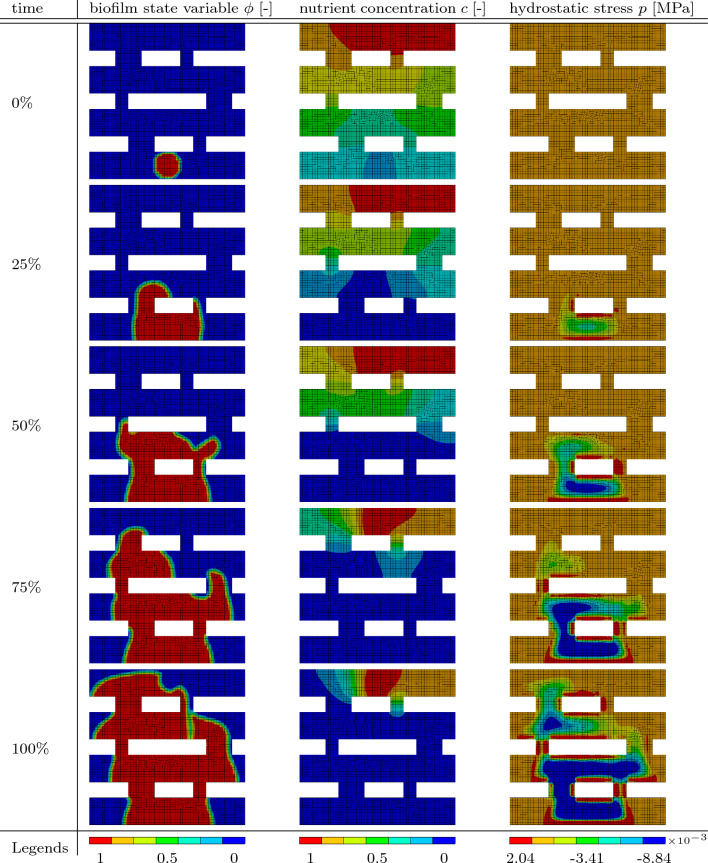
Results of the grate simulation. In the left column, the biofilm state variable $\phi$ is shown. In the middle column, the nutrient concentration *c* is presented at different times throughout the simulation. The right column shows the hydrostatic stress *p* at the corresponding simulation times

The geometry is a square grate that contains a starting population of biofilm on one end and nutrients on the other end. In Figure 12, the starting conditions can be observed. The evolution of the biofilm state variable $\phi$, the nutrient concentration *c*, and the hydrostatic stress *p* throughout the simulation is depicted in Table 8. From there, it can be seen that the biofilm is not strictly following just one path but rather splits up and recombines by the end of the simulation. In the last result for the biofilm state variable $\phi$ at $100\%$ of simulation time, a half-circle indent occurs at the top part of the biofilm. This is due to the fixed values of the nutrient concentration of $c = 1$ at these nodes. Since there is no gradient at the nodes, the biofilm has no incentive to grow there. The hydrostatic stress develops similarly to the previous boundary value problems. In the middle of the biofilm is an area of negative hydrostatic stress (pressure), while there is positive hydrostatic stress at the areas where the biofilm meets the boundary of the geometry.

**Fig. 13:**
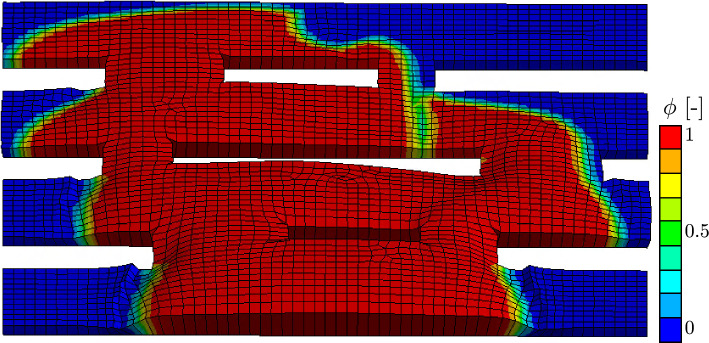
Oblique view of the field of the biofilm state variable $\phi$ of the grate boundary value problem. The distortion is enhanced by a factor of 500. Element swelling due to the evolution of $\alpha$ can be observed, especially in parts of the mesh where biofilm was present the longest

Figure 13 shows an oblique view of the boundary value problem at the end of the simulation.

The distortion of the elements is enhanced 500 times to better visualize the swelling of the biofilm due to the evolution of $\alpha$. It can be seen that not only does the biofilm state variable $\phi$ evolve through the domain, but also the domain itself changes in volume which results in the stresses. Parts of the mesh where biofilm is present continue to swell, indicating further growth by the biofilm living there. Since this swelling is accompanied by mesh distortion, only a certain amount of swelling can be computed before self-penetration, indicated by $\textrm{det}\, [\textbf{F}] < 0$, occurs and, consequently, the solver fails. This mesh distortion and subsequent failure of the solver, however, is not present in the numerical examples presented in this work.

## Conclusions

In this paper, we proposed a Hamilton-based growth model for biofilms that combines the volumetric approach based on the multiplicative split of deformation with a density-based approach. The former handles the volume change in the growth phenomenon, while the latter deals with the change (production) of mass as a result of the biological process. Using an extended Hamilton principle as the methodology for the derivation of the mathematical model, the first and second laws of thermodynamics are inherently fulfilled. It gives rise to the reliability, stability and robustness of the mathematical model. Several numerical test cases are provided to show the versatility of the presented model. Besides academic and classical examples, challenging and more exotic boundary condition problems are considered. The numerical results show a good agreement with the intuitive expectation as well as the available data in the literature. The numerical implementation is so stable that it is capable of capturing very complex morphology of biofilm in extremely irregular domains in the presence of numerous obstacles. It makes the model suitable for more realistic applications such as bioreactors in which intricate three-dimensional ducts and microchambers are designed to support bacterial growth and biological processes. Additionally, one can extend the model to incorporate different species of biofilms and their multilateral interactions. A preliminary investigation of a coaggregation between two-species systems has been performed in Soleimani et al. (2023) which can be generalized to an arbitrary number of species thanks to the Hamilton principle. This is necessary when it comes to the experimental validation of the model and is the direction of our future work. It is commonly known that real-world biofilms (for example those that inhabit the oral cavity or *in vivo* experiments) constitute hundreds of species and, hence, a decent numerical model must be as overarching as possible. Finally, the impact of antimicrobial substances (drugs) can be introduced into the model to have a comprehensive virtual platform for studying biofilms and the preventative measures to control them. This is also left for the last steps of our work.

## References

[CR1] Flemming H-C, Wingender J (2010) The biofilm matrix. Nat Rev Microbiol 8(9):623–633 20676145 10.1038/nrmicro2415

[CR2] Böl M, Ehret AE, Bolea Albero A, Hellriegel J, Krull R (2013) Recent advances in mechanical characterisation of biofilm and their significance for material modelling. Crit Rev Biotechnol 33(2):145–171 22642670 10.3109/07388551.2012.679250

[CR3] Nielsen PH, Jahn A, Palmgren R (1997) Conceptual model for production and composition of exopolymers in biofilms. Water Sci Technol 36(1):11–19

[CR4] Billings N, Birjiniuk A, Samad TS, Doyle PS, Ribbeck K (2015) Material properties of biofilms-a review of methods for understanding permeability and mechanics. Rep Progress Phys 78(3):036601 10.1088/0034-4885/78/3/036601 PMC4504244 25719969

[CR5] Soleimani M, Wriggers P, Rath H, Stiesch M (2016) Numerical simulation and experimental validation of biofilm in a multi-physics framework using an sph based method. Comput Mech 58(4):619–633

[CR6] Klapper I, Dockery J (2010) Mathematical description of microbial biofilms. SIAM Rev 52(2):221–265

[CR7] Soleimani M (2017) Numerical simulation and experimental validation of biofilm formation. PhD thesis

[CR8] Chinnaraj SB, Jayathilake PG, Dawson J, Ammar Y, Portoles J, Jakubovics N, Chen J (2021) Modelling the combined effect of surface roughness and topography on bacterial attachment. J Mater Sci Technol 81:151–161

[CR9] Albero AB, Ehret AE, Böl M (2014) A new approach to the simulation of microbial biofilms by a theory of fluid-like pressure-restricted finite growth. Comput Methods Appl Mech Eng 272:271–289

[CR10] Mattei M, Frunzo L, D’acunto B, Pechaud Y, Pirozzi F, Esposito G (2018) Continuum and discrete approach in modeling biofilm development and structure: a review. J Math Bio 76(4):945–1003 28741178 10.1007/s00285-017-1165-y

[CR11] Chaudhry MAS, Beg SA (1998) A review on the mathematical modeling of biofilm processes: advances in fundamentals of biofilm modeling. Chem Eng Technol Ind Chem Plant Equip Process Eng Biotechnol 21(9):701–710

[CR12] Soleimani M, Muthyala N, Marino M, Wriggers P (2020) A novel stress-induced anisotropic growth model driven by nutrient diffusion: theory, fem implementation and applications in bio-mechanical problems. J Mech Phys Solids 144:104097

[CR13] Goriely A (2017) The Mathematics and Mechanics of Biological Growth, vol 45. Springer, Berlin

[CR14] Epstein M, Maugin GA (2000) Thermomechanics of volumetric growth in uniform bodies. Int J Plast 16(7–8):951–978

[CR15] Lubarda VA, Hoger A (2002) On the mechanics of solids with a growing mass. Int J Solids Struct 39(18):4627–4664

[CR16] Haouala S, Doghri I (2015) Modeling and algorithms for two-scale time homogenization of viscoelastic-viscoplastic solids under large numbers of cycles. Int J Plast 70:98–125

[CR17] Hermanowicz SW (2001) A simple 2d biofilm model yields a variety of morphological features. Math Biosci 169(1):1–14 11137525 10.1016/s0025-5564(00)00049-3

[CR18] Lardon LA, Merkey BV, Martins S, Dötsch A, Picioreanu C, Kreft J-U, Smets BF (2011) idynomics: next-generation individual-based modelling of biofilms. Environ Microbiol 13(9):2416–2434 21410622 10.1111/j.1462-2920.2011.02414.x

[CR19] Li B, Taniguchi D, Gedara JP, Gogulancea V, Gonzalez-Cabaleiro R, Chen J, McGough AS, Ofiteru ID, Curtis TP, Zuliani P (2019) Nufeb: a massively parallel simulator for individual-based modelling of microbial communities. PLoS Comput Biol 15(12):1007125 10.1371/journal.pcbi.1007125 PMC6932830 31830032

[CR20] Naylor J, Fellermann H, Ding Y, Mohammed WK, Jakubovics NS, Mukherjee J, Biggs CA, Wright PC, Krasnogor N (2017) Simbiotics: a multiscale integrative platform for 3d modeling of bacterial populations. ACS Synth Biol 6(7):1194–1210 28475309 10.1021/acssynbio.6b00315

[CR21] Tack IL, Nimmegeers P, Akkermans S, Hashem I, Van Impe JF (2017) Simulation of escherichia coli dynamics in biofilms and submerged colonies with an individual-based model including metabolic network information. Front Microbiol 8:2509 29321772 10.3389/fmicb.2017.02509 PMC5733555

[CR22] Verhulst A, Cappuyns A, Van Derlinden E, Bernaerts K, Van Impe J (2011) Analysis of the lag phase to exponential growth transition by incorporating inoculum characteristics. Food Microbiol 28(4):656–666 21511125 10.1016/j.fm.2010.07.014

[CR23] Rath H, Feng D, Neuweiler I, Stumpp NS, Nackenhorst U, Stiesch M (2017) Biofilm formation by the oral pioneer colonizer *Streptococcus gordonii*: an experimental and numerical study. FEMS Microbiol Ecol 93(3):fix010 10.1093/femsec/fix010 28158402

[CR24] Rodriguez EK, Hoger A, McCulloch AD (1994) Stress-dependent finite growth in soft elastic tissues. J Biomech 27(4):455–467 8188726 10.1016/0021-9290(94)90021-3

[CR25] Soleimani M (2019) Finite strain visco-elastic growth driven by nutrient diffusion: theory, fem implementation and an application to the biofilm growth. Comput Mech 64(5):1289–1301

[CR26] Waffenschmidt T, Menzel A, Kuhl E (2012) Anisotropic density growth of bone-a computational micro-sphere approach. Int J Solids Struct 49(14):1928–1946

[CR27] Capriz G, Mariano PM (2003) Symmetries and hamiltonian formalism for complex materials. J Elast 72:57–70

[CR28] Junker P, Balzani D (2021) An extended hamilton principle as unifying theory for coupled problems and dissipative microstructure evolution. Continuum Mech Thermodyn 33(4):1931–1956

[CR29] Junker P, Wick T (2024) Space-time variational material modeling: a new paradigm demonstrated for thermo-mechanically coupled wave propagation, visco-elasticity, elasto-plasticity with hardening, and gradient-enhanced damage. Comput Mech 73(2):365–402

[CR30] Monod J (1949) The growth of bacterial cultures. Annu Rev Microbiol 3(1):371–394

[CR31] Flemming H-C, Wingender J (2001) Biofilme-die bevorzugte lebensform der bakterien: Flocken, filme und schlämme. Biol Unserer Zeit 31(3):169–180

[CR32] Chu EK, Kilic O, Cho H, Groisman A, Levchenko A (2018) Self-induced mechanical stress can trigger biofilm formation in uropathogenic *Escherichia coli*. Nat Commun 9(1):4087 30291231 10.1038/s41467-018-06552-z PMC6173693

[CR33] Korelc J, Wriggers P (2016) Automation of finite element methods. Springer, Berlin

[CR34] Hornung R, Grünberger A, Westerwalbesloh C, Kohlheyer D, Gompper G, Elgeti J (2018) Quantitative modelling of nutrient-limited growth of bacterial colonies in microfluidic cultivation. J R Soc Interface 15(139):20170713 29445038 10.1098/rsif.2017.0713 PMC5832723

[CR35] Toyofuku M, Inaba T, Kiyokawa T, Obana N, Yawata Y, Nomura N (2016) Environmental factors that shape biofilm formation. Biosci Biotechnol Biochem 80(1):7–12 26103134 10.1080/09168451.2015.1058701

[CR36] Soleimani M, Szafranski SP, Qu T, Mukherjee R, Stiesch M, Wriggers P, Junker P (2023) Numerical and experimental investigation of multi-species bacterial co-aggregation. Sci Rep 13(1):11839 37481628 10.1038/s41598-023-38806-2 PMC10363141

